# Supplementary material for: Joint COVID-19 and influenza-like illness forecasts in the United States using internet search information
Source: Commun Med (Lond). 2023 Mar 24;3:39. doi: 10.1038/s43856-023-00272-2 (PMC10038385; doi:10.1038/s43856-023-00272-2)
Supplement: Supplementary file 1 — Description of Additional Supplementary Files [file 43856_2023_272_MOESM1_ESM.pdf]

Description of Additional Supplementary Files

File Name: Supplementary Data 1

Description: Numerical data plotted in Figure 1
