# Supplementary material for: Joint COVID-19 and influenza-like illness forecasts in the United States using internet search information
Source: Commun Med (Lond). 2023 Mar 24;3:39. doi: 10.1038/s43856-023-00272-2 (PMC10038385; doi:10.1038/s43856-023-00272-2)
Supplement: Supplementary file 2 — Supplementary Information [file 43856_2023_272_MOESM2_ESM.pdf]

Supporting Information for Joint COVID-19 and Influenza-like Illness Forecasts in the United States using Internet Search Information

Simin Ma, Shaoyang Ning, Shihao Yang

Correspondence to: shihao.yang@isye.gatech.edu

Supplementary Methods

ARGO-Nat Prediction

COVID-19 Cases and Deaths

Here, we discuss the national-level COVID-19 cases and deaths prediction model in detail. On the high-level, we employ lagged (daily imputed) ILI information into "ARGO-Inspired" method [44] for both cases and deaths predictions, but with slight exogenous variable adjustments for cases predictions. Since the COVID-19 case trend leads the deaths trend not vise-versa, we include only COVID-19 lagged cases as time-series exogenous variables in cases prediction model, while using the same important Google search queries (with different optimal lags in the LASSO regression shown in table S3) and including lagged ILI information.

Let $X^C_{i,t,m}$ be the COVID-19 related Google Trends data of search term i day t of area m; $X^F_{i,\tau,m}$ be the Flu related Google Trends data of search term i week τ of area m; $y^D_{t,m}$ be the New York Times COVID-19 deaths at day t of area m; $y^C_{t,m}$ be the New York Times COVID-19 confirmed case at day t of area m; $y^H_{t,m}$ be the daily hospital admissions of region m on day t; $p^*_{\tau,m}$ be the CDC published %ILI for week τ of area m; $\bar{p}_{t,m}$ be the imputed (daily filled weekly number) %ILI for day t of area m, where the area m can refer to the entire nation, one specific HHS region (such as New England), or one specific state (such as Georgia). Let O^D_k be the optimal lag for the kth Google search term with respect to (w.r.p) COVID-19 deaths, and O^C_k be the optimal lag for the kth Google search term w.r.p to COVID-19 deaths, which are the same for all area m respectively. Let $\mathbb{I}_{\{t,r\}}$ be the weekday r indicator for t (i.e., $\mathbb{I}_{\{t,1\}}$ indicates day t being Monday, and $\mathbb{I}_{\{t,6\}}$ indicates day t being Saturday), which accounts for the weekday periodicity in COVID-19 deaths time series.

Inspired by ARGO method [22], with information available as of time T, to estimate $y^C_{T+l,m}$ and $y^D_{T+l,m}$ for $l > 0$, the COVID-19 cases and deaths on day $T + l$ of area m, L_1 regularized linear estimators are used:

$$\begin{aligned}\hat{y}^C_{T+l,m} = \hat{\mu}^C_{y,m} + \sum_{j\in\mathcal{C}^C} \hat{\beta}^C_{j,m} y^C_{T+l-j,m} + \sum_{k=1}^{K} \hat{\delta}^C_{k,m} X_{k,T+l-\hat{O}^C_k,m} + \sum_{r=1}^{6} \hat{\gamma}^C_{r,m}\mathbb{I}_{\{T+l,r\}} \\ + \sum_{h\in\mathcal{H}^C} \eta^C_{h,m}\bar{p}_{\mathcal{T}-h,m}\end{aligned} \tag{1}$$

$$\begin{aligned}\hat{y}^D_{T+l,m} = \hat{\mu}^D_{y,m} + \sum_{i=0}^{I} \hat{\alpha}^D_{i,m} y^D_{T-i,m} + \sum_{j\in\mathcal{J}^D} \hat{\beta}^D_{j,m} y^H_{T+l-j,m} + \sum_{k=1}^{K} \hat{\delta}^D_{k,m} X_{k,T+l-\hat{O}^D_k,m} + \sum_{r=1}^{6} \hat{\gamma}^D_{r,m}\mathbb{I}_{\{T+l,r\}} \\ + \sum_{h\in\mathcal{H}^D} \eta^D_{h,m}\bar{p}_{\mathcal{T}-h,m}\end{aligned} \tag{2}$$

We use lagged confirmed cases, cases' optimal lagged Google search terms and previous weeks' ILI for cases predictions. We use lagged deaths, lagged hospitalization, deaths' optimal lagged Google search terms and previous weeks' ILI for death prediction. For l^{th} day ahead cases predictions at area m, the coefficients of the case forecasting model $\boldsymbol{\theta}^C = \{\mu^C_{y,m}, \boldsymbol{\alpha}^C = (\alpha^C_{1,m}, \ldots, \alpha^C_{I,m}), \boldsymbol{\beta}^C = (\beta^C_{1,m}, \ldots, \beta^C_{|\mathcal{J}|,m}), \boldsymbol{\delta}^C = (\delta^C_{1,m}, \ldots, \delta^C_{K,m}), \boldsymbol{\gamma}^C = (\gamma^C_{1,m}, \ldots, \gamma^C_{6,m}), \boldsymbol{\eta}^C = \{\eta^C_{1,m}, \ldots, \eta^C_{|\mathcal{H}|,m}\}\}$ are obtained via

$$\begin{aligned}\underset{\boldsymbol{\theta}^C,\boldsymbol{\lambda}^C}{\text{argmin}} \sum_{t=T-M-l+1}^{T-l} \Bigg(y^C_{t+l,m} - \mu^C_{y,m} - \sum_{j\in\mathcal{J}^C} \beta^C_{j,m} y^C_{t+l-j,m} \\ - \sum_{k=1}^{27} \delta^C_{k,m} X_{k,t+l-\hat{O}^C_k,m} - \sum_{r=1}^{6} \gamma^C_{r,m}\mathbb{I}_{\{t+l,r\}} - \sum_{h\in\mathcal{H}^C} \eta^C_{h,m}\bar{p}_{\tau-1,m} \Bigg)^2 + \boldsymbol{\lambda}^C\|\boldsymbol{\theta}^C\|_1\end{aligned} \tag{3}$$

For l^{th} day ahead deaths predictions at area m, The coefficients of the deaths forecasting model $\boldsymbol{\theta}^D = \{\mu^D_{y,m}, \boldsymbol{\alpha}^D = (\alpha^D_{1,m}, \ldots, \alpha^D_{I,m}), \boldsymbol{\beta}^D = (\beta^D_{1,m}, \ldots, \beta^D_{|\mathcal{J}|,m}), \boldsymbol{\delta}^C = (\delta^D_{1,m}, \ldots, \delta^D_{K,m}), \boldsymbol{\gamma}^D = (\gamma^D_{1,m}, \ldots, \gamma^D_{6,m}), \boldsymbol{\eta}^D = \{\eta^D_{1,m}, \ldots, \eta^D_{|\mathcal{H}|,m}\}\}$ are obtained via

$$\begin{aligned}\underset{\boldsymbol{\theta}^D, \boldsymbol{\lambda}^D}{\operatorname{argmin}} \sum_{t=T-M-l+1}^{T-l} \Bigg(& y^D_{t+l,m} - \mu^D_{y,m} - \sum_{i=0}^{6} \alpha^D_{i,m} y^D_{t-i,m} - \sum_{j \in \mathcal{J}^D} \beta^D_{j,m} y^H_{t+l-j,m} \\ & - \sum_{k=1}^{27} \delta^D_{k,m} X_{k,t+l-\hat{O}^D_k,m} - \sum_{r=1}^{6} \gamma^D_{r,m} \mathbb{I}_{\{t+l,r\}} - \sum_{h \in \mathcal{H}^D} \eta^D_{h,m} \bar{p}_{\tau-1,m} \Bigg)^2 + \boldsymbol{\lambda}^D \|\boldsymbol{\theta}^D\|_1\end{aligned} \tag{4}$$

We set $M = 56$, i.e. 56 days as training period; $I = 6$ considering consecutive 1 week lagged target time series; $\mathcal{J}^D = \max(\{7, 14\}, l)$ considering weekly lagged confirmed hospitalization for death predictions, while $\mathcal{J}^C = \mathcal{J}^H = \max(\{7\}, l)$ considering only previous week's confirmed cases for case predictions; $K = 23$ highly correlated COVID-19 related Google search terms; $\hat{O}^C_k = \max\left(O^C_k, l\right)$ be the adjusted optimal lag w.r.p to COVID-19 cases, and $\hat{O}^D_k = \max\left(O^D_k, l\right)$ be the adjusted optimal lag w.r.p to COVID-19 deaths of kth Google search term subject to l^{th} day ahead prediction; $\mathcal{H}^D = \max(\{14, 21, 28\}, l)$ and $\mathcal{H}^C = \max(\{7, 14\}, l)$ considering weekly different lagged ILI for cases and deaths predictions. We set hyperparameters $\boldsymbol{\lambda}^C$ and $\boldsymbol{\lambda}^D$ through cross-validation separately.

To further impose smoothness into our predictions, we use the three-day moving average of the coefficients for predicting day $T + l$, which slightly boosts our prediction accuracy.

Using the above formulation, we forecast future 4 weeks of daily COVID-19 cases and deaths of area m, i.e. $\{\hat{y}^C_{T+1,m}, \ldots, \hat{y}^C_{T+28,m}\}$ and $\{\hat{y}^D_{T+1,m}, \ldots, \hat{y}^D_{T+28,m}\}$, and aggregate them into weekly predictions. In other words, $\hat{y}^C_{T+1:T+7,m} = \sum_{i=1}^{7} \hat{y}^C_{T+i,m}$ is first week's prediction, and $\hat{y}^C_{T+8:T+14,m} = \sum_{i=8}^{14} \hat{y}^C_{T+i,m}$ is the second week's prediction and etc, similarly for weekly deaths predictions. We denote this method as "ARGO Inspired Prediction".

%ILI

We obtain an accurate estimate $\hat{p}^*_{\tau,m}$ for the %ILI for week $\tau = \{1, \ldots, \mathcal{T}\}$ of area m using the ARGO method [22], additionally incorporating area m consecutive 1 week lagged COVID-19 cases as exogenous variables.

Newly Proposed Bi-disease ARGOX-Local (Step 2 in Figure 2)

Since %ILI and COVID-19 cases and deaths have strong connections between each state and its neighbours (Figure 1), this section introduces a modified ARGOX framework [24, 44], by incorporating spatial temporal flu information for COVID-19 cases/deaths state level estimates, and considering COVID-19 cases information for %ILI estimates, while treating each state separately to produce individual state-level forecasts.

For the state m, our raw estimates for its weekly COVID-19 cases/deaths $y_{\tau,m}$ are $\hat{y}^{GT}_{\tau,m}$, $\hat{y}^{reg}_{\tau,r_m}$, $\hat{y}^{nat}_{\tau}$ and $\bar{p}_{\tau-1,m}$, where r_m is the region number for state m, and $\bar{p}_{\tau-1,m}$ is the weekly aggregated daily imputed ILI of week $\tau-1$ in state m (since ILI lag one week behind COVID-19 published data). Here, we overload the notations without superscript specifying COVID-19 cases (C) and deaths (D), as their predictions' frameworks are the same. Similar to the second step in ARGOX [24], we denote the deaths increment at week τ of state m as $Z_{\tau,m} = \Delta y_{\tau,m} = y_{\tau,m} - y_{\tau-1,m}$ (target scalar) and it has the following predictors: (i) $Z_{\tau-1,m} = \Delta y_{\tau-1,m}$, (ii) $\{\hat{y}^{GT}_{\tau,m} - y_{\tau-1,m}\}_{m\in\mathcal{M}}$, (iii) $\hat{y}^{reg}_{\tau,m} - y_{\tau-1,m}$, (iv) $\hat{y}^{nat}_{\tau} - y_{\tau-1,m}$, and (v) $\{\bar{p}_{\tau-1,m} - \bar{p}_{\tau-2,m}\}_{m\in\mathcal{M}}$, where $\mathcal{M}$ is a set containing all states in the same region as the target state m. In other words, there are $(2|\mathcal{M}|+3)$ predictors for state m, and let's denote the predictor vector as $\boldsymbol{W}_{\tau,m} = (Z_{\tau-1,m}, \{\hat{y}^{GT}_{\tau,m} - y_{\tau-1,m}\}_{m\in\mathcal{M}}, (\hat{y}^{reg}_{\tau} - y_{\tau-1,m}), (\hat{y}^{nat}_{\tau} - y_{\tau-1,m}), \{\bar{p}_{\tau-1,m} - \bar{p}_{\tau-2,m}\}_{m\in\mathcal{M}})^{\intercal}$, which follows a VAR-X (vector autoregressive with exogenous variables) structure with $Z_{\tau-1}$ serving as the autoregressive lagged 1 term and the rest serving as exogenous variables.

We propose a modified structured variance-covariance matrix for reliable estimation and numerical stability (see subsection below). Lastly, the best linear predictor with ridge-regression inspired shrinkage [24] is used to obtain the estimate for each state (the VAR-X structure $\boldsymbol{W}_{\tau,m}$ above), and we take the median across these 100 estimations as our final predictions, considering 30 weeks training period.

Future %ILI predictions also follow the similar framework as above, incorporating neighbouring state's COVID-19 cases information as the additional predictor in the second step of ARGOX [24]. Particularly, our raw estimates for $p^*_{\tau,m}$, the %ILI of state m week τ, are $\hat{p}^{GT}_{\tau,m}$ (from ARGOX first step [24]), $\hat{p}^{reg}_{\tau,r_m}$ (from ARGO2 first step [23]), $\hat{p}^{nat}_{\tau,m}$ (from ARGO [22]), and $y^C_{\tau,m}$ (weekly aggregated COVID-19 cases of week τ in state m from NYT), where r_m is the region number for state m. Similarly, we denote the predictor vector for our target $Z^F_{\tau,m} = \Delta p^*_{\tau,m} = p^*_{\tau,m} - p^*_{\tau-1,m}$, as $\boldsymbol{W}^F_{\tau,m} = (Z^F_{\tau-1}, \{\hat{p}^{GT}_{\tau,m} - p^*_{\tau-1,m}\}_{m\in\mathcal{M}}, (\hat{p}^{reg}_{\tau,r_m} - p^*_{\tau-1,m}), (\hat{p}^{nat}_{\tau} - p^*_{\tau-1,m}), \{y^C_{\tau,m} - y^C_{\tau-1,m}\}_{m\in\mathcal{M}})^{\intercal}$, where F stands for "Flu". Since NYT publishes daily COVID-19 reported cases, we are able to use week τ's COVID-19 cases when prediction week τ's %ILI. Lastly, the best linear predictor with ridge-regression inspired shrinkage [24] is used to get the final estimate.

Structured Variance-Covariance Matrix

Here, we illustrate the proposed structured variance-covariance matrix and its assumption used in ARGOX-Local, through COVID-19 cases/deaths prediction. %ILI prediction also follows the same framework.

The predictor for COVID-19 cases/deaths' increment $Z_{\tau,m} = y_{\tau,m} - y_{\tau-1,m}$ at week τ in state m is $\boldsymbol{W}_{\tau,m} = (Z_{\tau-1,m}, \{\hat{y}^{GT}_{\tau,m} - y_{\tau-1,m}\}_{m\in\mathcal{M}}, (\hat{y}^{reg}_{\tau} - y_{\tau-1,m}), (\hat{y}^{nat}_{\tau} - y_{\tau-1,m}), \{\bar{p}_{\tau-1,m} - \bar{p}_{\tau-2,m}\}_{m\in\mathcal{M}})^{\intercal}$. Let's denote the nth imputed week τ ILI increment in state m as $Z^F_{\tau,m} = \{\bar{p}_{\tau,m} - \bar{p}_{\tau-1,m}\}_{m\in\mathcal{M}}$.

From ARGOX [24], the final best linear predictor with ridge-regression-inspired shrinkage for $Z_{\tau,m}$ is

$$\hat{Z}_{\tau,m} = \mu^{(m)}_Z + \frac{1}{2}\Sigma^{(m)}_{ZW}(\frac{1}{2}\Sigma^{(m)}_{WW} + \frac{1}{2}D^{(m)}_{WW})^{-1}(\boldsymbol{W}_{\tau,m} - \boldsymbol{\mu}^{(m)}_W). \tag{5}$$

where $\mu^{(m)}_Z$ and $\boldsymbol{\mu}^{(m)}_W$ are the mean scalar and vector of $Z_{\tau,m}$ and $\boldsymbol{W}_{\tau,m}$ respectively. $\sigma^{2^{(m)}}_{ZZ}$, $\Sigma^{(m)}_{ZW}$, $\Sigma^{(m)}_{WW}$ are covariance matrix of and between $Z_{\tau,m}$ and $\boldsymbol{W}_{\tau,m}$. The best linear predictor gives the optimal way to linearly combine the four predictors to form a new one.

We structure the covariance matrices for reliable estimation, following the same assumptions in ARGOX [24]:

1. The time series increments of COVID-19 cases/deaths and ILI are stationary and have a stable auto-correlation and cross-correlation across time and between COVID-19 and flu. Therefore, the covariances between the time series increments for state m satisfy
   - $\text{var}(Z_{\tau,m}) = \text{var}(Z_{\tau-1,m}) = \sigma^{2^{(m)}}_{ZZ}$
   - $\text{cov}(\boldsymbol{Z}_\tau, \boldsymbol{Z}_{\tau-1}) = \rho^{(m)}\sigma^{2^{(m)}}_{ZZ}$
   - $var(Z^F_{\tau,m}) = var(Z^F_{\tau-1,m}) = \sigma^{2^{(m)}}_{FF}$

- $cov(Z_\tau, Z_\tau^F) = \sigma_{ZF}^{2^{(m)}}$
- $cov(Z_\tau, Z_{\tau-1}^F) = \rho_F^{(m)} \sigma_{ZF}^{2^{(m)}}$.

2. Different sources of information are independent for COVID and Flu for a given state m. In other words, $Z_{\tau,m}, \hat{y}_{\tau,m}^{GT} - y_{\tau,m}, \hat{y}_{\tau,m}^{reg} - y_{\tau,m}, \hat{y}_{\tau,m}^{nat} - y_{\tau,m}, Z_{\tau,m}^F$ are mutually independent (where the boldface represents full vectors containing all the states).

The covariance matrices are thereby simplified as:

$$\Sigma_{ZW}^{(m)} = \begin{pmatrix} \rho^{(m)}\sigma_{ZZ}^{2^{(m)}} & \sigma_{ZZ}^{2^{(m)}} & \sigma_{ZZ}^{2^{(m)}} & \sigma_{ZZ}^{2^{(m)}} & \rho_F^{(m)}\sigma_{ZF}^{2^{(m)}} \end{pmatrix} \tag{6}$$

$$\Sigma_{WW} = \begin{pmatrix} \sigma_{ZZ}^{2^{(m)}} & \{\rho^{(m)}\sigma_{ZZ}^{2^{(m)}}\}_{m\in\mathcal{M}} & \rho^{(m)}\sigma_{ZZ}^{2^{(m)}} & \rho^{(m)}\sigma_{ZZ}^{2^{(m)}} & \{\sigma_{ZF}^{2^{(m)}}\}_{m\in\mathcal{M}} \\ \{\rho^{(m)}\sigma_{ZZ}^{2^{(m)}}\}_{m\in\mathcal{M}}^\intercal & \Sigma_{ZZ}^{\mathcal{M}} + \Sigma_{\mathcal{M}}^{GT} & \sigma_{ZZ}^{2^{(m)}} & \sigma_{ZZ}^{2^{(m)}} & \{\rho_F^{(m)}\sigma_{ZF}^{2^{(m)}}\}_{m\in\mathcal{M}} \\ \rho^{(m)}\sigma_{ZZ}^{2^{(m)}} & \sigma_{ZZ}^{2^{(m)}} & \sigma_{ZZ}^{2^{(m)}} + \sigma^{2^{reg}} & \sigma_{ZZ}^{2^{(m)}} & \{\rho_F^{(m)}\sigma_{ZF}^{2^{(m)}}\}_{m\in\mathcal{M}} \\ \rho^{(m)}\sigma_{ZZ}^{2^{(m)}} & \sigma_{ZZ}^{2^{(m)}} & \sigma_{ZZ}^{2^{(m)}} & \sigma_{ZZ}^{2^{(m)}} + \sigma^{2^{nat}} & \{\rho_F^{(m)}\sigma_{ZF}^{2^{(m)}}\}_{m\in\mathcal{M}} \\ \{\sigma_{ZF}^{2^{(m)}}\}_{m\in\mathcal{M}}^\intercal & \{\rho_F^{(m)}\sigma_{ZF}^{2^{(m)}}\}_{m\in\mathcal{M}}^\intercal & \{\rho_F^{(m)}\sigma_{ZF}^{2^{(m)}}\}_{m\in\mathcal{M}}^\intercal & \{\rho_F^{(m)}\sigma_{ZF}^{2^{(m)}}\}_{m\in\mathcal{M}}^\intercal & \Sigma_{FF}^{\mathcal{M}} \end{pmatrix} \tag{7}$$

where $\Sigma_{ZZ}^{\mathcal{M}} = \text{var}(Z_{\tau,m\in\mathcal{M}})$, $\Sigma_{FF}^{\mathcal{M}} = \text{var}(Z_{\tau,m\in\mathcal{M}}^F)$ $\Sigma_{\mathcal{M}}^{reg} = \text{var}(\hat{y}_{\tau,m\in\mathcal{M}}^{reg} - y_{\tau,m\in\mathcal{M}})$, $\Sigma_{\mathcal{M}}^{nat} = \text{var}(\hat{y}_{\tau,m\in\mathcal{M}}^{nat} - y_{\tau,m\in\mathcal{M}})$, and $\Sigma_{\mathcal{M}}^{GT} = \text{var}(\hat{y}_{\tau,m\in\mathcal{M}}^{GT} - y_{\tau,m\in\mathcal{M}})$.

Note that $\mu_Z^{(m)}, \boldsymbol{\mu}_W^{(m)}, \sigma_{ZZ}^{2^{(m)}}, \sigma_{ZF}^{2^{(m)}}, \Sigma_{ZZ}^{\mathcal{M}}, \Sigma_{FF}^{\mathcal{M}}, \Sigma_{\mathcal{M}}^{reg}, \Sigma_{\mathcal{M}}^{nat}, \Sigma_{\mathcal{M}}^{GT}$ are estimated by the corresponding sample mean and covariance from the data in the most recent 2-month training window.

Thus, our final prediction for state m at week τ COVID-19 week τ is

$$\hat{y}_{\tau,m} = \hat{y}_{\tau-1,m} + \mu_Z^{(m)} + \Sigma_{ZW}^{(m)}(\Sigma_{WW}^{(m)} + D_{WW}^{(m)})^{-1}(\boldsymbol{W}_{\tau,m} - \boldsymbol{\mu}_W^{(m)}). \tag{8}$$

To obtain the prediction interval of $\hat{y}_{\tau,m}$, we first estimate the variance of the prediction residual empirically. In particular, we estimate $\text{Var}(y_{\tau,m} - \hat{y}_{\tau,m}|y_{\tau-1,m}, y_{\tau-2,m}, \ldots)$ using the most recent 2-month training window's empirical residuals. Then, the corresponding $1-\alpha\%$ prediction interval estimate is as follows:

$$\hat{y}_{\tau,m} \pm z_{1-\alpha}\sqrt{\text{Var}(y_{\tau,m} - \hat{y}_{\tau,m}|y_{\tau-1,m}, y_{\tau-2,m}, \ldots)} \tag{9}$$

Modifications of Previously-Proposed Single-Disease Methods (Step 2 in Figure 2) and ARGOX-Joint-Ensemble (Step 3 in Figure 2)

This section explains the modifications of previously-proposed single-disease COVID-19 and Influenza-like Illness forecasting models incorporated in ARGOX-Joint-Ensemble framework (Step 2 in Figure 2).

For COVID-19 predictions, we combine single-disease Ref [44] (ARGOX) and newly proposed bi-disease ARGOX-Local (above) into the ensemble forecast, ARGOX-Joint-Ensemble. As Ref [44] is initially proposed as a COVID-19 deaths forecasting model, there are slight modifications when we are targeting COVID-19 cases. Specifically, single-disease Ref [44] is also an ensemble model combined three sub-models: ARGO (similar to ARGO-Nat but with no ILI information), ARGOX-2Step, and ARGOX-NatConstraint. Below, we state those modifications for ARGOX-2Step and ARGOX-NatConstraint.

For %ILI predictions, we combine single-disease Ref [24] and newly proposed bi-disease ARGOX-Local (above) into the ensemble forecast. There are no modifications needed as we are not switching targets here.

ARGOX-2Step

Here, ARGOX-2Step operates slightly differently for cases and deaths predictions. For COVID-19 deaths forecasts, similar to previous study [44], the second step takes a dichotomous approach for the joint states and alone states, identified through geographical separations and multiple correlations on COVID-19 deaths growth trends, where the joint states are forecast jointly utilizing cross-state, cross-source information from each other, while alone states are forecasted separately each with its own state and national information. On the other hand, we unify all the states and forecast jointly for cases predictions. The reason behind different second step approaches in the ARGOX framework lies in the sparsity of state-level COVID-19 cases and deaths data. COVID-19 deaths are sparse and not well correlated with other states and regions for those isolated (alone) states, which weakens the impact from cross-state, cross-region information in the joint framework. Meanwhile, COVID-19 cases are dense and well-correlated cross state and regions, which indicate advantages of using joint estimations.

Lastly, we estimate 1-2 weeks ahead state-level %ILI using ARGOX [24].

ARGOX-NatConstraint

Similar to prior study, we incorporate a constrained second step to ARGOX inspired state level prediction above [44] for both COVID-19 cases and deaths forecasts, while separating out HI and VT during deaths forecasting, and no such separation in cases forecasting, due to data sparsity.

ARGOX-Joint-Ensemble

Here, we further explain the final ensemble step (third step in Figure 2) stated in the Methods section. To further boost the state-level COVID-19 and %ILI prediction accuracy, we incorporate an ensemble framework that combines our previous estimations and selects the best predictor for each week.

For all 51 U.S. states' COVID-19 cases/deaths as targets, we have four predictions from Step 2 (Figure 2): "ARGO", "ARGOX-2Step", "ARGOX-Nat-Constraint", and "ARGOX-Local", where the first three are single-disease sub-models introduced in Ref [44] and bi-disease "ARGOX-Local" is proposed in this study. Here, we want to state that we are careful on the indexing and are not using any forward looking information, when we use the sub-models to obtain COVID-19 cases/deaths estimates in Step 2 (Figure 2). As an example, "standing on" August 13 2022, to produce 1-4 weeks ahead forecasts (8/20/2022, 8/27/2022, 9/3/2022, 9/10/2022), we use COVID-19 cases, deaths, hospitalization, and Google search data up to August 13 2022, and ILI data up to August 5 2022 (since CDC published the %ILI reports for the past week every Friday). For a training period of **overlapping 15 weeks**, we evaluate each predictor with mean squared error (MSE) and select the one with lowest MSE as the ensemble predictor for the forecast horizon of interest. For example, "standing on" August 13, 2022, the 15 weeks' 1-week-ahead forecasts (training data) are 8/13/2022, 8/12/2022, ... , 7/30/2022, where each training estimate is a rolling weekly estimate stored in daily index. Daily and weekly indexing details are further explained in the section below. Therefore, the overlapping 15 weeks training period is roughly a 2-week time span. The overlapping 15 weeks' forecasts seem to be highly correlated, but this generates better performances than using 2 separate weeks' forecasts for MSE comparison and ensemble selection (i.e. keep all raw and sub-model estimates in weekly frequencies). Also, note that we flattened out "ARGO", "ARGOX-2Step", and "ARGOX-Nat-Constraint" in the final ensemble step

here to produce a 4-method ensemble forecast as ARGOX-Joint-Ensemble's final forecast, due to simplicity. The results are similar if we conduct a 2-method ensemble forecast, i.e. not flattening the 3 single-disease sub-models introduced in Ref [44].

For all 51 U.S. regions' %ILI as targets, we have two predictions: bi-disease ARGOX-Local and single-disease ARGOX (Ref [24]). For example, "standing on" August 12, 2022, to produce 1-2 weeks ahead forecasts (8/12/2022 and 8/19/2022), we use COVID-19 cases and %ILI related Google search frequency available up to August 11, 2022, and %ILI available up to August 5, 2022. Since CDC releases a report of %ILI for the previous week every Friday, by the time they release the %ILI report on August 12, 2022 (stores weekly %ILI from August 6, 2022 to August 12, 2022), one already know COVID-19 and Google search information up to August 11, 2022. We take the advantage of the 1-week-lag of CDC reporting to produce 1 and 2 weeks ahead %ILI forecasts. For a training period of **non-overlapping** 15 weeks, we evaluate each predictor with MSE and select the one with lowest MSE as the ensemble predictor for week $(\tau + 1)$ and $(\tau + 2)$. For example, "standing on" August 12, 2022, the 15 weeks' 1-week-ahead forecasts (training data) are 8/12/2022, 8/5/2022, 7/29/2022, ..., 5/6/2022.

Lastly, ARGOX-Joint-Ensemble's prediction interval for state m at week τ directly corresponds to the selected method's prediction interval in the state and week of interest. The detailed descriptions of the "ARGO", "ARGOX-2Step", and "ARGOX-Nat-Constraint" prediction interval calculations can be found in Ref [44]. On the other hand, "ARGOX-Local" prediction interval is computed empirically, and the details are described in the section "Newly Proposed Bi-disease ARGOX-Local" above. As an example, if ARGOX-Joint-Ensemble selected "ARGOX-Local" as the "best" predictor for week 8/20/2022 in Georgia, the prediction interval will be calculated by "ARGOX-Local".

Daily and weekly indexing details

Note that this only occur when the forecast target is COVID-19 cases and deaths. There are no such indexing cross-over in %ILI forecasts. Since COVID-19 cases, deaths and related Google search queries are all in daily frequencies, it is intuitive to produce daily forecasts. Thus, for national-level, we use the "ARGO-Nat" method to produce 28-day-ahead daily forecasts, and aggregate them into weekly forecasts to compare with other publicly available methods (following CDC Forecast Hub guidelines [34]). Similarly, on the state-level, in Step 1 (Figure 2), all the raw estimates of COVID-19 cases/deaths are first obtained in daily frequencies and aggregated into weekly estimates. We essentially store them as rolling weekly estimates stored in the daily index. For example, on date August 13, 2022, all raw estimates store the weekly estimate from August 7, 2022 to August 13, 2022; on date August 12, 2022, they store the weekly estimate from August 6, 2022 to August 12, 2022, etc. By doing this, we are enabled with more data, which is especially helpful for early stage COVID-19 predictions. Thus, all four sub-models (Step 2 2) also contain weekly estimates but daily indexing.

Supplementary Tables

	Location with Detail Description
National-Level	
Single-disease ARGO Method	Ref [44]
Bi-disease ARGO-Nat Method	Supplementary Materials:<br>Section “ARGO-Nat Prediction”
State-Level	
Single-disease “ARGO” Method	Ref [44]
Single-disease “ARGOX-2Step” Method	Ref [44] and Supplementary Materials:<br>Section “ARGOX-2Step”
Single-disease “ARGOX-NatConstraint” Method	Ref [44] and Supplementary Materials:<br>Section “ARGOX-NatConstraint”
Bi-disease “ARGOX-Local” Method	Supplementary Materials:<br>Section “Newly Proposed Bi-disease ARGOX-Local”
ARGOX-Joint-Ensemble Method	Supplementary Materials:<br>Section “ARGOX-Joint-Ensemble”

Table S1: Method’s Naming Convention with Target COVID-19 Cases/Deaths. The table includes details on COVID-19 forecasting methods’ abbreviated names and locations of more detailed descriptions.

	Location with Detail Description
National-Level	
Single-disease ARGO Method	Ref [22]
Bi-disease ARGO-Nat Method	Supplementary Materials: Section “ARGO-Nat Prediction”
State-Level	
Single-disease “ARGOX” Method	Ref [24]
Bi-disease “ARGOX-Local” Method	Supplementary Materials:<br>Section “Newly Proposed Bi-disease ARGOX-Local”
ARGOX-Joint-Ensemble Method	Supplementary Materials: Section “ARGOX-Joint-Ensemble”

Table S2: Method’s Naming Convention with Target %ILI. The table includes details on the %ILI forecasting methods’ abbreviated names and locations of more detailed descriptions.

Google Search Term	Optimal Lag for COVID-19 Cases	Optimal Lag for COVID-19 Deaths
coronavirus vaccine	4	5
cough	4	24
covid 19 vaccine	34	7
coronavirus exposure	5	13
coronavirus cases	35	30
coronavirus test	35	30
covid 19 cases	35	30
covid 19	34	21
exposed to coronavirus	6	24
fever	4	24
headache	5	29
how long covid 19	6	21
how long contagious	6	25
loss of smell	10	25
loss of taste	11	24
nausea	10	23
pneumonia	34	28
rapid covid 19	8	28
rapid coronavirus	8	27
robitussin	35	15
sore throat	8	30
sinus	8	15
symptoms of the covid 19	16	21

Table S3: The selected 23 important terms' optimal lags. The optimal lags are selected through fitting regression of lagged terms against COVID-19 cases and deaths (seperately) and select the ones with minimal mean-squared error. The deriving period is from April 1st 2020 to June 30th 2020.

	1 Week Ahead	2 Weeks Ahead	3 Weeks Ahead	4 Weeks Ahead
COVID-19 Cases				
ARGO	28.4%	26.2%	31.5%	36.1%
ARGOX-2Step	19.2%	20.2%	23.8%	25.1%
ARGOX-NatConstraint	17.2%	18.5%	19.1%	19.8%
ARGOX-Local	36.2%	38.1%	25.6%	19.0%
COVID-19 Deaths				
ARGO	28.4%	27.8%	30.5%	32.9%
ARGOX-2Step	18.2%	19.1%	21.2%	21.1%
ARGOX-NatConstraint	13.1%	15.5%	17.1%	18.8%
ARGOX-Local	41.3%	39.6%	32.2%	29.2%
%ILI				
ARGOX	53.9%	51.8%		
ARGOX-Local	46.1%	48.2%		

Table S4: ARGOX-Joint-Ensemble selections of sub-models, across all U.S. states/area for 1 to 4 weeks ahead predictions for COVID-19 cases and deaths, and 1-2 weeks ahead prediction for %ILI. For COVID-19 Cases/Deaths selections, "ARGO", "ARGOX-2Step", and "ARGOX-NatConstraint" are single-disease sub-models introduced in Ref [44], and bi-disease "ARGOX-Local" is the method developed in this study. For %ILI selections, "ARGOX" is introduced in Ref [24].

	1 Week Ahead	2 Weeks Ahead	3 Weeks Ahead	4 Weeks Ahead	Average
From 2020-11-14 to 2021-03-06					
ARGOX-Joint-Ensemble	**111.301**	**145.946**	162.109	169.740	**147.274**
COVIDhub-ensemble[34]	117.941	151.295	**160.400**	**165.204**	148.710
Naive	121.147	154.608	178.312	188.493	160.640
From 2021-07-31 to 2021-11-06					
ARGOX-Joint-Ensemble	**61.780**	**73.157**	93.247	117.853	86.509
COVIDhub-ensemble[34]	62.680	75.236	**84.767**	**103.116**	**81.449**
Naive	65.923	89.062	109.188	129.451	98.325
From 2022-01-01 to 2022-03-19					
ARGOX-Joint-Ensemble	**90.544**	129.303	151.269	183.407	138.630
COVIDhub-ensemble[34]	94.014	**128.650**	**147.171**	**172.914**	**135.687**
Naive	103.092	132.665	157.165	180.430	143.338

Table S5: Comparison among different models' 1 to 4 weeks ahead U.S. states level weekly deaths predictions in three rapidly changing dynmics: COVID-19 second wave (2020-11-14 to 2021-03-06), COVID-19 Delta variant led wave (2021-07-31 to 2021-11-06), and COVID-19 Omicron variant led wave (2022-01-01 to 2022-03-19). The RMSE and their averages over 51 states are reported. The best performing method is boldfaced.

	1 Week Ahead	2 Weeks Ahead	3 Weeks Ahead	4 Weeks Ahead	Average
From 2020-10-31 to 2021-02-20					
ARGOX-Joint-Ensemble	**5340.54**	**8724.51**	9207.23	13482.54	9188.70
COVIDhub-ensemble[34]	5502.03	8935.14	**9177.59**	**11540.55**	**8788.83**
Naive	5771.50	9068.55	11514.31	13683.07	10009.36
From 2021-07-17 to 2021-10-23					
ARGOX-Joint-Ensemble	**3121.52**	**5262.91**	8124.68	10003.79	6628.22
COVIDhub-ensemble[34]	3141.14	5390.01	**7804.07**	**8951.37**	**6321.64**
Naive	3441.26	5752.55	8023.08	9964.55	6795.36
From 2021-12-18 to 2022-03-05					
ARGOX-Joint-Ensemble	**20919.12**	42689.05	65876.74	72497.11	50495.51
COVIDhub-ensemble[34]	21532.51	**42003.17**	61771.91	68314.98	**48405.64**
Naive	25902.99	44421.40	**58205.82**	**66891.56**	48855.44

Table S6: Comparison among different models' 1 to 4 weeks ahead U.S. states level weekly cases predictions in three rapidly changing dynmics: COVID-19 second wave (2020-10-31 to 2021-02-20), COVID-19 Delta variant led wave (2021-07-17 to 2021-10-23), and COVID-19 Omicron variant led wave (2021-12-18 to 2022-03-05). The RMSE and their averages over 51 states are reported. The best performing method is boldfaced.

	1 Week Ahead	2 Weeks Ahead	3 Weeks Ahead	4 Weeks Ahead	Average
WIS					
COVIDhub-ensemble[34]	41.92	49.51	53.80	54.55	49.94
LANL-GrowthRate [47]	41.23	41.88	62.75	64.92	52.69
MOBS-GLEAM COVID [46]	41.38	52.45	59.23	68.23	55.32
UMass-MechBayes[45]	42.44	55.43	63.24	75.90	59.25
ARGOX-Joint-Ensemble	(#1) 40.45	(#3)52.12	(#5)69.82	(#6)80.23	(#5)60.65
UA-EpiCovDA [48]	46.29	54.94	71.01	78.41	62.66
epiforecasts-ensemble1 [49]	58.83	155.03	144.02	167.83	131.42
Coverage					
UMass-MechBayes[45]	0.90	0.91	0.89	0.88	0.89
COVIDhub-ensemble[34]	0.89	0.90	0.88	0.86	0.88
ARGOX-Joint-Ensemble	(#1)0.91	(#3)0.88	(#3)0.85	(#3)0.80	(#3)0.86
LANL-GrowthRate [47]	0.85	0.82	0.81	0.77	0.81
epiforecasts-ensemble1 [49]	0.87	0.78	0.78	0.76	0.79
MOBS-GLEAM COVID [46]	0.78	0.63	0.64	0.63	0.67
UA-EpiCovDA [48]	0.72	0.63	0.60	0.62	0.64

Table S7: State-Level Death Prediction Interval Comparisons among different models' 1-4 weeks ahead COVID-19 deaths (from 2020-07-04 to 2022-08-03). The weighted interval score (WIS), coverage and their averages are reported. The reported WIS and coverage for each forecast horizon are averaged across all 51 regions. Methods are sorted based on their average. Our ARGOX-Joint-Ensemble's ranking for each error metric are included in parenthesis. The WIS is evaluated across $K = 11$ prediction intervals with $\alpha_1 = 0.02$, $\alpha_2 = 0.05$, $\alpha_3 = 0.1, \ldots, \alpha_{11} = 0.9$ (implying nominal coverages of 98%, 95%, 90%, …, 10%) [34, 55].

	1 Week Ahead	2 Weeks Ahead	3 Weeks Ahead	4 Weeks Ahead	Average
WIS					
UVA-Ensemble[51]	3661.74	6014.52	6494.23	7388.82	5889.83
COVIDhub-ensemble[34]	3679.22	5832.72	7294.78	7323.23	6032.49
Karlen-pypm[54]	3923.60	6023.20	7284.59	7723.09	6238.62
CovidAnalytics-DELPHI[53]	4211.11	5736.61	7584.51	8215.93	6437.04
ARGOX-Joint-Ensemble	(#3)3692.81	(#3)5825.35	(#6)7971.04	(#7)8283.04	(#5)6443.06
USC-SI_kJalpha[50]	4506.92	5719.51	7019.69	8236.22	6470.58
CU-select[52]	4385.70	7257.43	7977.58	7933.29	6888.50
Coverage					
COVIDhub-ensemble[34]	0.88	0.85	0.77	0.73	0.81
UVA-Ensemble[51]	0.87	0.80	0.72	0.61	0.75
ARGOX-Joint-Ensemble	(#2)0.88	(#2)0.80	(#4)0.60	(#6)0.52	(#3)0.70
CU-select[52]	0.73	0.67	0.63	0.59	0.65
Karlen-pypm[54]	0.78	0.70	0.59	0.50	0.64
CovidAnalytics-DELPHI[53]	0.60	0.59	0.59	0.58	0.59
USC-SI_kJalpha[50]	0.53	0.50	0.57	0.53	0.53

Table S8: State-Level Case Prediction Interval Comparisons among different models' 1-4 weeks ahead COVID-19 cases (from 2020-07-04 to 2022-08-03). The weighted interval score (WIS), coverage and their averages are reported. The reported WIS and coverage for each forecast horizon are averaged across all 51 regions. Methods are sorted based on their average. Our ARGOX-Joint-Ensemble's ranking for each error metric are included in parenthesis.

	1 Week Ahead	2 Weeks Ahead	Average
WIS			
ARGOX-Joint-Ensemble	**0.36**	**0.41**	**0.38**
VAR	0.45	0.55	0.50
Coverage			
ARGOX-Joint-Ensemble	**0.93**	**0.89**	**0.91**
VAR	0.88	0.78	0.83

Table S9: State-Level %ILI Prediction Interval Comparisons among different models' 1-2 weeks ahead %ILI (from 2020-07-04 to 2022-08-03). The weighted interval score (WIS), coverage and their averages are reported. The reported WIS and coverage for each forecast horizon are averaged across all 51 regions. Best performed method is highlighted in boldface. VAR: lag-1 vector autoregressive model)

	1 Week Ahead	2 Weeks Ahead	3 Weeks Ahead	4 Weeks Ahead
RMSE				
Naive	2068.825	2900.719	3769.794	4574.886
ARGO-Nat (prev. impute)	**1785.597**	**1797.031**	2895.129	3803.325
ARGO-Nat (cur. impute)	1787.691	1800.781	**2843.261**	**3795.023**
MAE				
Naive	1460.341	2143.057	2943.011	3598.909
ARGO-Nat (prev. impute)	1251.489	**1284.782**	**1990.523**	2692.364
ARGO-Nat (cur. impute)	**1240.861**	1296.206	2001.850	**2680.633**
Correlation				
Naive	0.930	0.863	0.769	0.656
ARGO-Nat (prev. impute)	0.951	**0.955**	0.898	**0.852**
ARGO-Nat (cur. impute)	**0.954**	0.952	**0.901**	0.842

Table S10: National Level Deaths Prediction Comparison between different ILI imputation methods and Naive in 3 error metrics, during the period from 2020-07-04 to 2022-03-05. Boldface highlights the best performance for each metric in each study period, for 1-4 weeks ahead predictions. All comparisons are based on the original scale of COVID-19 national deaths. "prev. impute": imputes daily ILI from weekly ILI using COVID-19 daily cases fluctuation within weekdays. "cur. impute" imputes daily ILI as the same as weekly ILI.

	1 Week Ahead	2 Weeks Ahead	3 Weeks Ahead	4 Weeks Ahead
RMSE				
Naive	2002.163	2843.254	3785.285	4716.175
Ref [44]	1574.677	3125.644	3513.936	4597.786
ARGO-Nat	**1460.687**	**2881.785**	**3329.274**	**4531.994**
MAE				
Naive	1388.912	2049.515	2875.619	3659.958
Ref [44]	1127.963	1774.636	2363.010	**3071.537**
ARGO-Nat	**1086.401**	**1620.623**	**2185.010**	3080.635
Correlation				
Naive	0.940	0.880	0.789	0.676
Ref [44]	0.963	0.898	0.840	0.825
ARGO-Nat	**0.964**	**0.908**	**0.873**	**0.841**

Table S11: National Level Deaths Prediction Comparison in 3 Error Metrics. Boldface highlights the best performance for each metric in each study period, for 1-4 weeks ahead predictions. All comparisons are based on the original scale of COVID-19 national deaths. On average, ARGO-Nat is able to achieve around 5.5% RMSE, 5% MAE reduction, and around 1.5% correlation improvement from previously proposed ARGO Inspired method [44].

	1 Week Ahead	2 Weeks Ahead	3 Weeks Ahead	4 Weeks Ahead	Average
RMSE					
COVIDhub-ensemble [34]	1391.95	1757.32	2256.52	2797.28	2050.77
LANL-GrowthRate [47]	1669.58	2209.95	2818.20	3800.58	2624.58
UA-EpiCovDA [48]	1987.54	2578.35	3147.84	3558.53	2818.07
ARGOX-Joint-Ensemble	(#2)1460.68	(#6)2881.78	(#4)3329.27	(#5)4532.00	(#4)3050.93
epiforecasts-ensemble1 [49]	2340.40	2872.52	3474.88	4236.03	3230.95
Naive	2002.16	2843.25	3785.28	4716.17	3336.71
MOBS-GLEAM_COVID [46]	2412.44	3282.19	4171.40	4981.60	3711.91
UMass-MechBayes [45]	1677.23	2624.11	4171.37	6388.44	3715.29
MAE					
COVIDhub-ensemble [34]	981.88	1233.60	1553.79	1967.95	1434.31
UMass-MechBayes [45]	1121.31	1500.15	2120.60	2800.88	1885.74
UA-EpiCovDA [48]	1372.78	1720.42	2092.43	2766.98	1988.15
ARGOX-Joint-Ensemble	(#2)1086.40	(#3)1620.62	(#5)2185.01	(#5)3080.63	(#4)1993.16
LANL-GrowthRate [47]	1239.30	1702.86	2129.45	2920.92	1998.13
Naive	1388.91	2049.51	2875.61	3659.95	2493.50
epiforecasts-ensemble1 [49]	1539.70	1951.13	2409.16	3079.42	2244.85
MOBS-GLEAM_COVID [46]	1656.30	2364.67	3196.92	3932.89	2787.69
Correlation					
COVIDhub-ensemble[34]	0.97	0.95	0.92	0.87	0.93
ARGOX-Joint-Ensemble	(#2)0.96	(#3)0.91	(#3)0.87	(#2)0.84	(#2)0.90
LANL-GrowthRate[47]	0.96	0.93	0.88	0.80	0.89
UA-EpiCovDA[48]	0.95	0.90	0.84	0.79	0.87
UMass-MechBayes[45]	0.95	0.90	0.80	0.71	0.84
epiforecasts-ensemble1[49]	0.91	0.87	0.81	0.73	0.83
Naive	0.94	0.88	0.79	0.67	0.82
MOBS-GLEAM_COVID[46]	0.91	0.83	0.73	0.63	0.77

Table S12: National Level Death Prediction Comparisons among different models' 1 to 4 weeks ahead weekly deaths (from 2020-07-04 to 2022-08-13). The RMSE, MAE, Pearson correlation and their averages are reported. Methods are sorted based on their average. Our ARGOX-Joint-Ensemble's ranking for each error metric are included in parenthesis.

	1 Week Ahead	2 Weeks Ahead	3 Weeks Ahead	4 Weeks Ahead
RMSE				
Naive	383651.1	682473.2	916564.9	**1083258.2**
Ref [44]	290364.1	596468.4	883279.2	1201914.0
ARGO-Nat	**262456.8**	**582431.2**	**864164.7**	1208269.0
MAE				
Naive	178138.9	307726.8	426259.1	**525528.0**
Ref [44]	122804.4	249293.4	407772.6	561084.6
ARGO-Nat	**115248.8**	**248746.7**	**406912.8**	578659.5
Correlation				
Naive	0.910	0.715	0.487	0.285
Ref [44]	0.973	0.850	0.510	0.348
ARGO-Nat	**0.977**	**0.879**	**0.636**	**0.389**

Table S13: National Level Case Prediction Comparison in 3 Error Metrics. Boldface highlights the best performance for each metric in each study period. All comparisons are based on the original scale of COVID-19 national deaths. On average, ARGO-Nat is able to achieve around 5% RMSE, 6.5% MAE reduction and 3% Correlation increase from previously proposed ARGO Inspired method [44]

	1 Week Ahead	2 Weeks Ahead	3 Weeks Ahead	4 Weeks Ahead	Average
RMSE					
CU-select[52]	266727.69	434455.22	653538.38	801585.50	539076.70
UVA-Ensemble[51]	453112.20	674208.16	810552.03	907374.65	711311.76
Karlen-pypm[54]	411376.15	655077.02	843095.27	1005935.37	728870.95
ARGOX-Joint-Ensemble	(#1)262456.84	(#1)582431.21	(#5)864164.73	(#7)1208269.03	(#4)729330.40
CovidAnalytics-DELPHI[53]	490793.09	672264.08	829104.25	929606.87	730442.07
COVIDhub-ensemble[34]	273140.06	673459.73	907201.30	1071743.89	731386.24
Naive	383651.15	682473.25	916564.90	1083258.26	766486.89
USC-SI_kJalpha[50]	384489.33	1018298.54	1770603.69	1998695.41	1293021.74
MAE					
CU-select[52]	134621.57	206681.54	305180.50	379034.75	256379.59
UVA-Ensemble[51]	195414.46	286828.81	374178.39	430499.91	321730.40
COVIDhub-ensemble[34]	124424.38	283266.72	401587.76	495779.84	326264.67
ARGOX-Joint-Ensemble	(#1)115248.81	(#2)248746.73	(#4)406912.82	(#7)588659.55	(#4)337392.34
Naive	178138.95	307726.80	426259.16	525528.03	359413.24
Karlen-pypm[54]	178335.71	297506.37	424294.34	552867.54	363250.99
USC-SI_kJalpha[50]	147195.37	320727.67	517725.17	629338.36	403746.64
CovidAnalytics-DELPHI[53]	275737.21	369334.32	458784.19	517622.59	405369.57
Correlation					
USC-SI_kJalpha[50]	0.98	0.93	0.81	0.64	0.84
CU-select[52]	0.96	0.89	0.73	0.55	0.78
ARGOX-Joint-Ensemble	(#2) 0.97	(#3) 0.88	(#3) 0.66	(#5) 0.38	(#3) 0.72
Karlen-pypm[54]	0.91	0.75	0.56	0.40	0.65
CovidAnalytics-DELPHI[53]	0.89	0.74	0.54	0.40	0.64
COVIDhub-ensemble[34]	0.96	0.73	0.51	0.32	0.63
UVA-Ensemble[51]	0.87	0.67	0.50	0.35	0.60
Naive	0.91	0.72	0.49	0.29	0.60

Table S14: National Level Case Prediction Comparisons among different models' 1 to 4 weeks ahead weekly cases (from 2020-07-04 to 2022-08-13). The RMSE, MAE, Pearson correlation and their averages are reported. Methods are sorted based on their average. Our ARGOX-Joint-Ensemble's ranking for each error metric are included in parenthesis.

	1 Week Ahead	2 Weeks Ahead
RMSE		
Naive	0.208	0.373
Ref [22]	0.152	0.245
ARGO-Nat	**0.138**	0.222
AR-3	0.177	0.710
MAE		
Naive	0.124	0.217
Ref [22]	0.107	0.149
ARGO-Nat	**0.100**	0.140
AR-3	0.108	0.518
Correlation		
Naive	0.954	0.853
Ref [22]	0.977	0.935
ARGO-Nat	**0.978**	0.947
AR-3	0.967	0.283

Table S15: National Level %ILI 1-2 weeks ahead Prediction Comparison in 3 Error Metrics. Boldface highlights the best performance for each metric in each study period. All comparisons are based on the original scale of CDC published %ILI. ARGO-Nat is able to achieve 9% RMSE, 6.2% MAE and 0.7% increase in correlation from ARGO method [22].

	1 Week Ahead	2 Weeks Ahead	3 Weeks Ahead	4 Weeks Ahead
RMSE				
ARGOX-Joint-Ensemble	**74.13**	90.14	**129.83**	**187.42**
• Ref [44]	79.40	**88.83**	133.83	195.62
• ARGOX-Local	99.39	129.44	195.78	245.95
Naive	106.20	147.86	199.52	250.76
MAE				
ARGOX-Joint-Ensemble	**52.55**	70.74	**72.26**	**110.62**
• Ref [44]	59.39	**68.93**	79.07	119.31
• ARGOX-Local	70.36	96.47	128.51	191.47
Naive	82.26	116.96	158.00	201.55
Correlation				
ARGOX-Joint-Ensemble	**0.96**	0.92	**0.90**	0.83
• Ref [44]	0.95	**0.94**	0.89	0.81
• ARGOX-Local	0.92	0.87	0.88	**0.88**
Naive	0.91	0.81	0.66	0.45

Table S16: Comparison of different methods for state-level COVID-19 1 to 4 weeks ahead death in Georgia (GA), from 2020-07-04 to 2022-08-13. The MSE, MAE, and correlation are reported and best performed method is highlighted in boldface.

	1 Week Ahead	2 Weeks Ahead	3 Weeks Ahead	4 Weeks Ahead	Average
WIS					
COVIDhub-ensemble[34]	30.95	32.23	**33.51**	40.24	**34.23**
ARGOX-Joint-Ensemble	**28.45**	**32.12**	39.28	49.83	37.42
Coverage					
COVIDhub-ensemble[34]	0.93	0.91	**0.90**	**0.86**	**0.90**
ARGOX-Joint-Ensemble	**0.96**	**0.91**	0.87	0.81	0.88

Table S17: Georgia's Death Prediction Interval Comparisons among COVIDhub-ensemble [34] and ARGOX-Joint-Ensemble, for 1-4 weeks ahead COVID-19 deaths (from 2020-07-04 to 2022-08-03). The weighted interval score (WIS), coverage and their averages are reported. The best performing method is boldfaced.

	1 Week Ahead	2 Weeks Ahead	3 Weeks Ahead	4 Weeks Ahead
RMSE				
ARGOX-Joint-Ensemble	**197.86**	**188.80**	223.56	**208.54**
• Ref [44]	236.14	226.44	224.96	211.62
• ARGOX-Local	214.87	227.25	**209.67**	237.71
Naive	245.77	255.54	272.12	241.45
MAE				
ARGOX-Joint-Ensemble	**80.62**	**79.77**	**101.75**	**104.41**
• Ref [44]	93.15	91.57	104.95	108.01
• ARGOX-Local	94.64	128.23	142.86	163.89
Naive	98.43	127.38	157.87	164.91
Correlation				
ARGOX-Joint-Ensemble	0.49	**0.57**	**0.82**	0.53
• Ref [44]	**0.78**	0.46	0.82	0.53
• ARGOX-Local	0.45	0.52	0.61	**0.55**
Naive	0.34	0.29	0.18	0.20

Table S18: Comparison of different methods for state-level COVID-19 1 to 4 weeks ahead death in North Carolina (NC), from 2020-07-04 to 2022-08-13. The MSE, MAE, and correlation are reported and best performed method is highlighted in boldface.

	1 Week Ahead	2 Weeks Ahead	3 Weeks Ahead	4 Weeks Ahead	Average
WIS					
COVIDhub-ensemble[34]	45.23	**49.90**	**49.30**	**56.81**	**50.31**
ARGOX-Joint-Ensemble	**43.59**	50.10	59.38	70.12	55.79
Coverage					
COVIDhub-ensemble[34]	0.92	**0.92**	0.90	0.86	**0.90**
ARGOX-Joint-Ensemble	**0.95**	0.91	0.85	0.79	0.87

Table S19: North Carolina's Death Prediction Interval Comparisons among COVIDhub-ensemble [34] and ARGOX-Joint-Ensemble, for 1-4 weeks ahead COVID-19 deaths (from 2020-07-04 to 2022-08-03). The weighted interval score (WIS), coverage and their averages are reported. The best performing method is boldfaced

	1 Week Ahead	2 Weeks Ahead	3 Weeks Ahead	4 Weeks Ahead
RMSE				
ARGOX-Joint-Ensemble	**14168.49**	**28273.03**	**34202.03**	47710.33
• Ref [44]	19568.58	34176.94	38105.01	49270.35
• ARGOX-Local	15158.35	32746.50	40573.39	50979.72
Naive	16376.03	29114.77	36740.58	**44754.24**
MAE				
ARGOX-Joint-Ensemble	**6810.10**	**15132.36**	**17952.78**	29152.97
• Ref [44]	8075.88	17719.45	20662.41	31894.74
• ARGOX-Local	9003.88	17846.60	30491.45	38564.03
Naive	8824.37	15260.00	21699.00	**27784.41**
Correlation				
ARGOX-Joint-Ensemble	**0.92**	0.85	0.88	0.83
• Ref [44]	0.86	0.84	**0.90**	**0.86**
• ARGOX-Local	0.91	**0.88**	0.44	0.18
Naive	0.90	0.73	0.47	0.21

Table S20: Comparison of different methods for state-level COVID-19 1 to 4 weeks ahead cases in Georgia (GA), from 2020-07-04 to 2022-08-13. The MSE, MAE, and correlation are reported and best performed method is highlighted in boldface.

	1 Week Ahead	2 Weeks Ahead	3 Weeks Ahead	4 Weeks Ahead
RMSE				
ARGOX-Joint-Ensemble	**17415.41**	**24907.07**	**41920.17**	60086.56
• Ref [44]	17651.51	33132.87	49445.76	61685.31
• ARGOX-Local	17495.78	37963.34	50796.31	66113.17
Naive	20468.31	36551.17	49285.13	**58001.50**
MAE				
ARGOX-Joint-Ensemble	9155.10	**13688.12**	**20547.07**	**30453.56**
• Ref [44]	**8077.13**	14598.05	24700.06	34380.86
• ARGOX-Local	9340.31	17410.98	28046.01	46856.69
Naive	10691.83	19324.08	26904.88	33263.45
Correlation				
ARGOX-Joint-Ensemble	**0.94**	**0.91**	**0.93**	**0.89**
• Ref [44]	0.94	0.87	0.89	0.89
• ARGOX-Local	0.94	0.77	0.44	0.21
Naive	0.90	0.68	0.41	0.19

Table S21: Comparison of different methods for state-level COVID-19 1 to 4 weeks ahead cases in North Carolina (NC), from 2020-07-04 to 2022-08-13. The MSE, MAE, and correlation are reported and best performed method is highlighted in boldface.

	1 Week Ahead	2 Weeks Ahead
RMSE		
ARGOX-Joint-Ensemble	**0.22**	**0.41**
• Ref [24]	0.30	0.45
• ARGOX-Local	0.28	0.43
Naive	0.33	0.57
VAR	0.48	0.69
MAE		
ARGOX-Joint-Ensemble	**0.13**	**0.36**
• Ref [24]	0.21	0.39
• ARGOX-Local	0.19	0.37
Naive	0.23	0.52
VAR	0.32	0.58
Correlation		
ARGOX-Joint-Ensemble	**0.98**	**0.91**
• Ref [24]	0.92	0.90
• ARGOX-Local	0.93	0.91
Naive	0.90	0.88
VAR	0.84	0.80

Table S22: Comparison of different methods for state-level %ILI 1 and 2 weeks ahead estimations in Georgia (GA), from 2020-07-04 to 2022-08-13. The MSE, MAE, and correlation are reported and best performed method is highlighted in boldface. The plot shows the entire evaluation period, from 2020-07-04 to 2022-08-13.

	1 Week Ahead	2 Weeks Ahead
RMSE		
ARGOX-Joint-Ensemble	**0.09**	**0.23**
• Ref [24]	0.10	0.27
• ARGOX-Local	0.11	0.25
Naive	0.17	0.32
VAR	0.13	0.36
MAE		
ARGOX-Joint-Ensemble	**0.05**	**0.15**
• Ref [24]	0.08	0.17
• ARGOX-Local	0.07	0.15
Naive	0.10	0.24
VAR	0.10	0.25
Correlation		
ARGOX-Joint-Ensemble	**0.98**	**0.93**
• Ref [24]	0.97	0.92
• ARGOX-Local	0.98	0.93
Naive	0.97	0.91
VAR	0.91	0.90

Table S23: Comparison of different methods for state-level %ILI 1 and 2 weeks ahead estimations in North Carolina (NC), from 2020-07-04 to 2022-08-13. The MSE, MAE, and correlation are reported and best performed method is highlighted in boldface.

Supplementary Figures

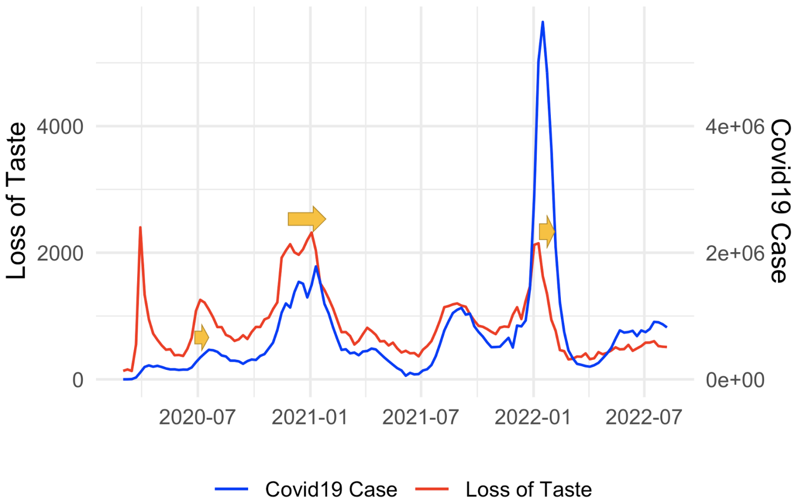

(a) National COVID-19 weekly cases and query “loss of taste” search frequencies.

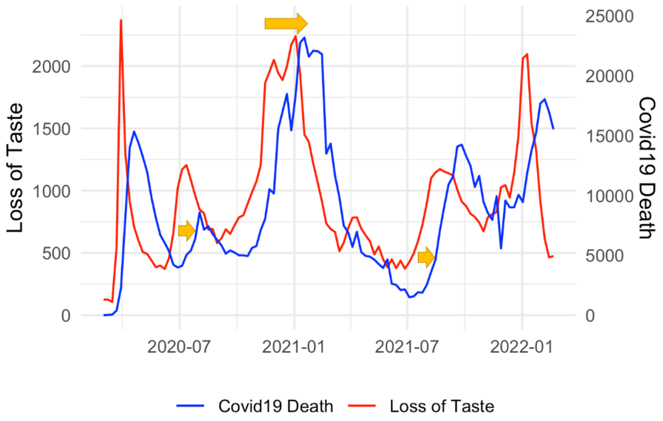

(b) National COVID-19 weekly deaths and query “loss of taste” search frequencies.

Figure S1: Google search query “loss of taste” and COVID-19 weekly cases (a) and deaths (b). Google search frequencies “loss of taste” is in red, and COVID-19 U.S. national level weekly cases and deaths are in blue. Y-axis are adjusted accordingly. This figure illustrates the delay in peak between the search frequency of “loss of taste” to COVID-19 cases and deaths.

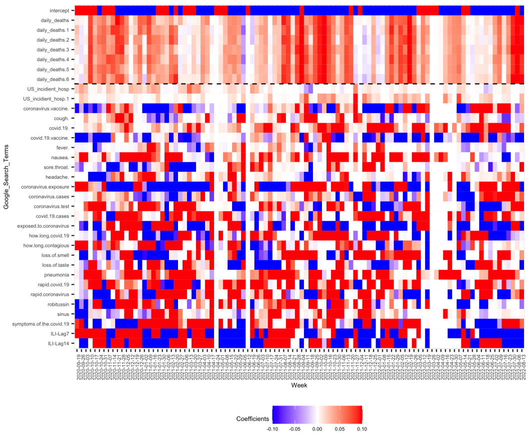

(a) 1 Week Ahead Coefficients

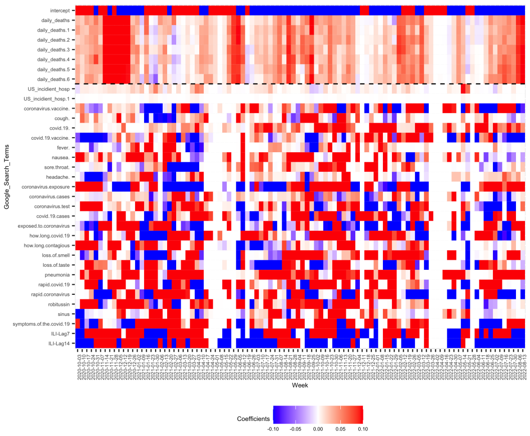

(b) 2 Week Ahead Coefficients

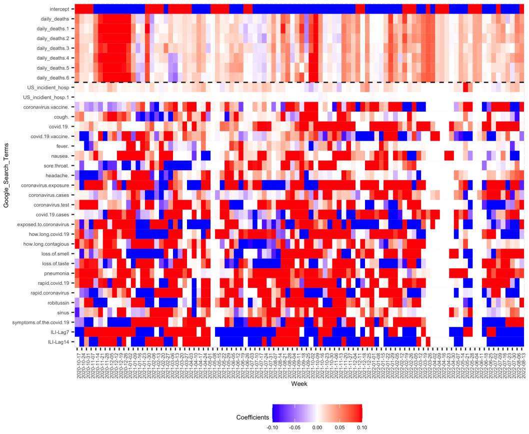

(c) 3 Week Ahead Coefficients

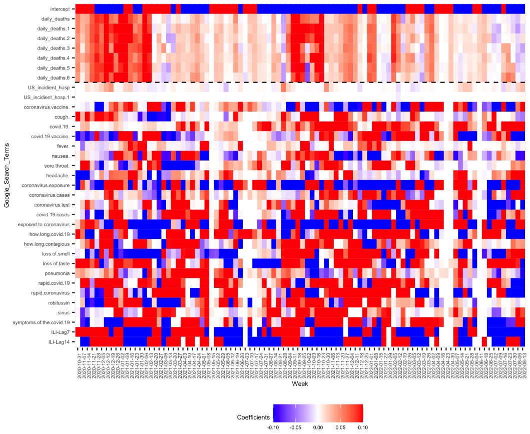

(d) 4 Week Ahead Coefficients

Figure S2: Smoothed coefficients for ARGO national level 1-4 weeks ahead predictions for COVID-19 Deaths. Coefficients larger than 0.1 are scaled to 0.1 and lower than -0.1 are scaled to -0.1, for simplicity. Red and blue represent positive and negative coefficients. Black horizontal dashed line separates Google query queries from autoregressive lags. The past 4 weeks %ILI information as exogenous variables are at the bottom 4 rows.

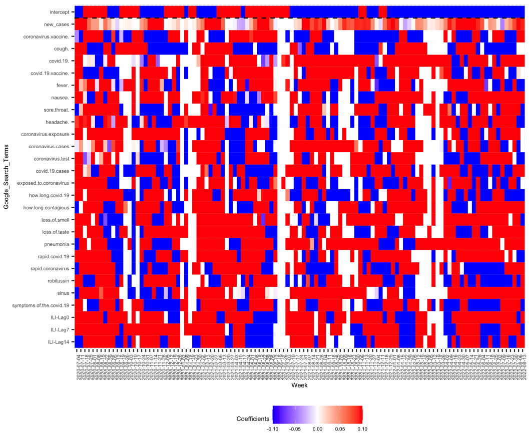

(a) 1 Week Ahead Coefficients

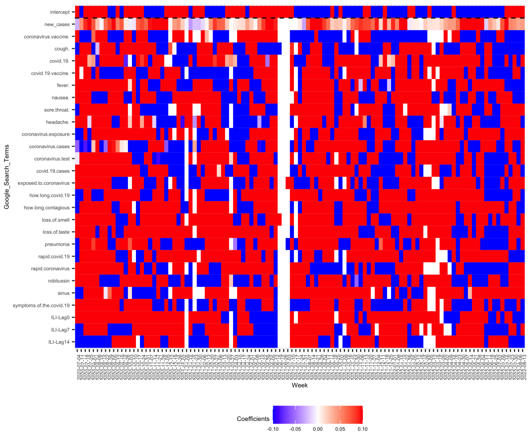

(b) 2 Weeks Ahead Coefficients

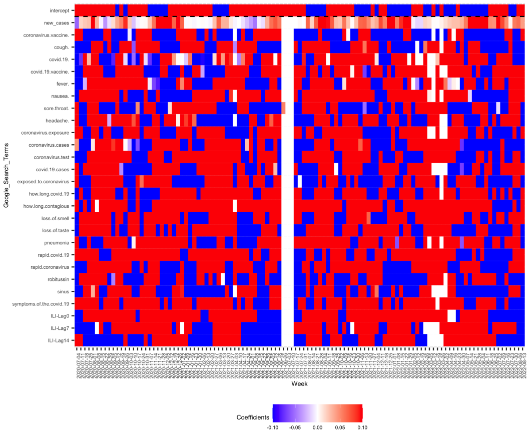

(c) 3 Weeks Ahead Coefficients

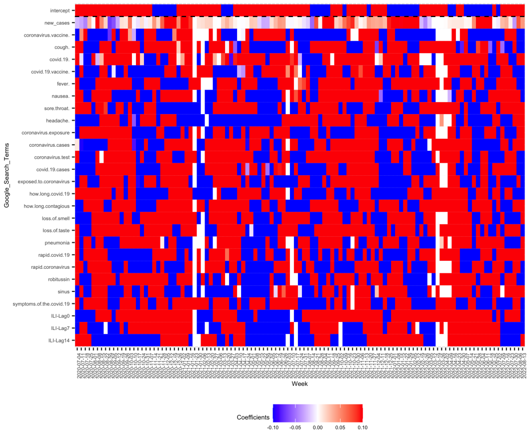

(d) 4 Weeks Ahead Coefficients

Figure S3: Smoothed coefficients for ARGO national level 1-4 weeks ahead predictions for COVID-19 Cases. Coefficients larger than 0.1 are scaled to 0.1 and lower than -0.1 are scaled to -0.1, for simplicity. Red and blue color represent positive and negative coefficients. Black horizontal dashed line separates Google query queries from autoregressive lags. The past 4 weeks %ILI information as exogenous variables are at the bottom.

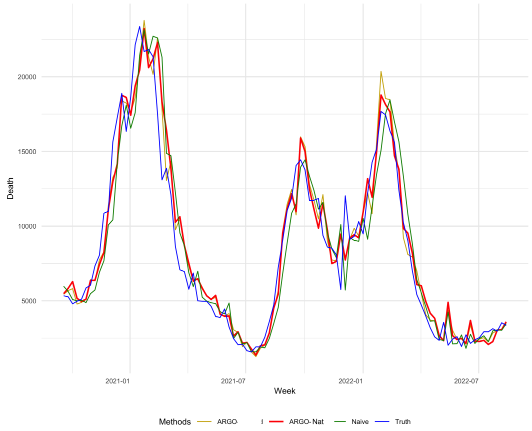

(a) 1 Week Ahead National Level Predictions

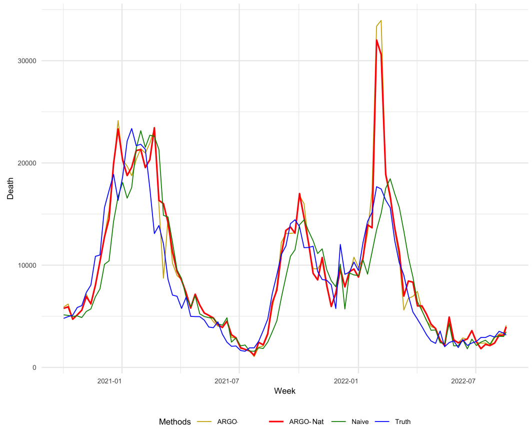

(b) 2 Weeks Ahead National Level Predictions

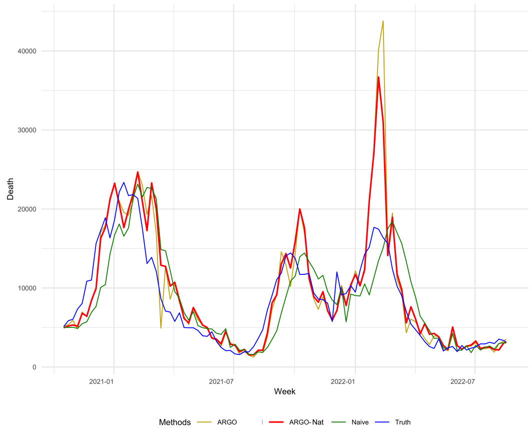

(c) 3 Weeks Ahead National Level Predictions

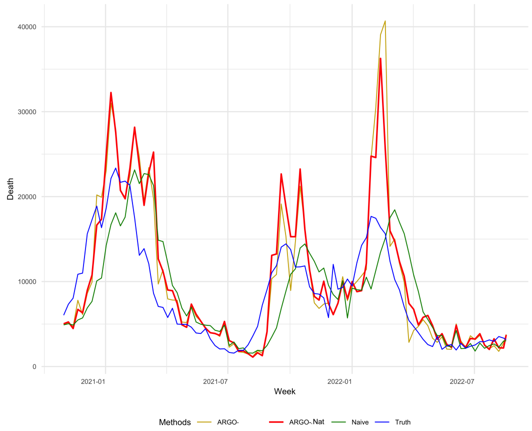

(d) 4 Weeks Ahead National Level Predictions

Figure S4: National level 1 to 4 weeks ahead COVID-19 weekly death predictions. The method included are single-disease ARGO (Ref [44]), bi-disease ARGO-Nat (see Methods Section), Naive (persistence), truth, weekly from 2020-07-04 to 2022-08-13. Estimation results for 1 (top left), 2 (top right), 3 (bottom left), and 4 (bottom right) weeks ahead weekly deaths. ARGO-Nat estimations (thick red), contrasting with the true COVID-19 deaths from JHU dataset (blue) as well as the estimates from Ref [44] (gold) and Naive (green).

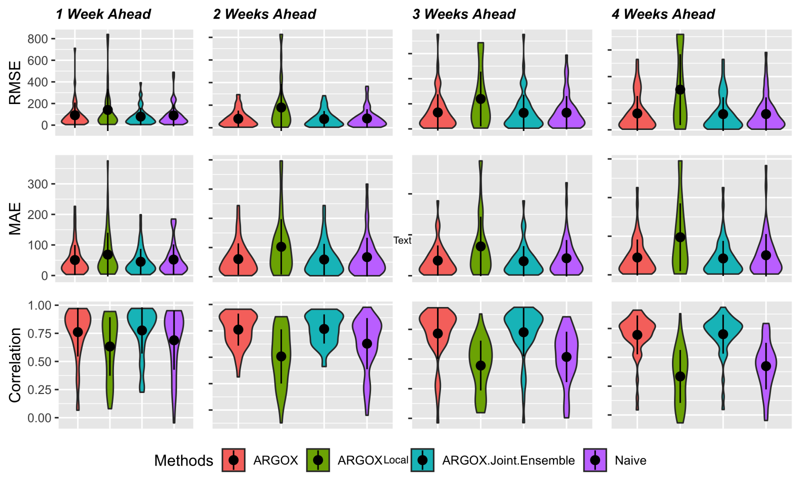

Figure S5: State Level Deaths Prediction Comparison in 3 Error Metrics. Comparison among different versions of our models' 1 to 4 weeks (from left to right) ahead U.S. states level weekly deaths predictions (from 2020-07-04 to 2022-08-13). The RMSE, MAE and Pearson correlation for each method across all states are reported in the violin plot. The methods (x-axis) are sorted based on their RMSE. Here, ARGOX denotes the previously proposed method, Ref [44].

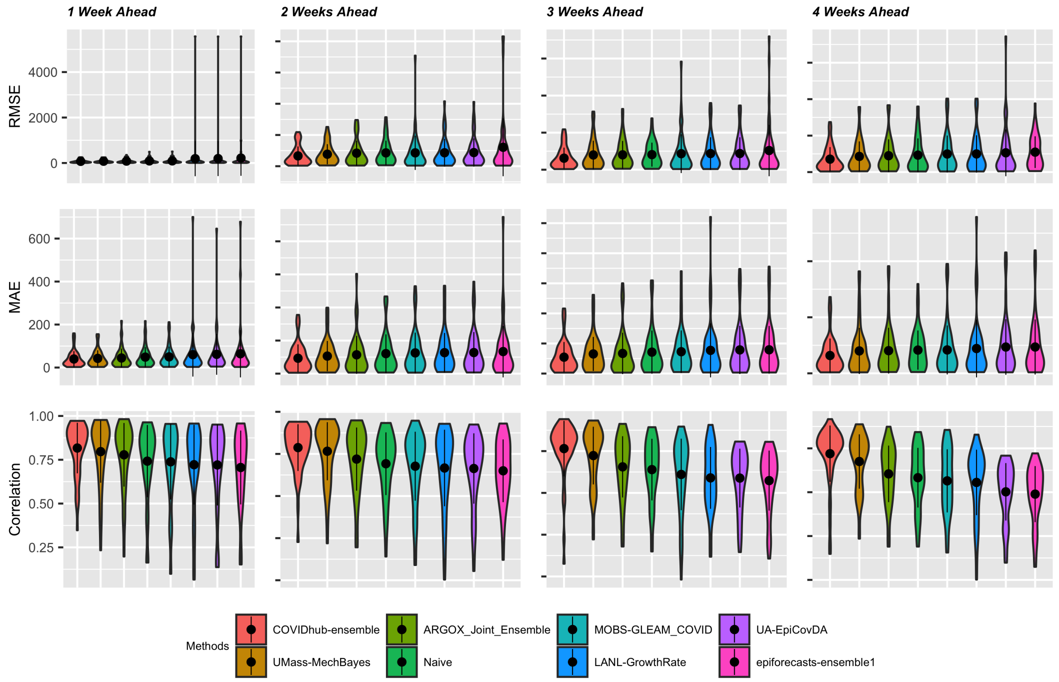

Figure S6: State Level Deaths Prediction Comparisons among different CDC published teams' 1 to 4 weeks (from left to right) ahead weekly deaths (from 2020-07-04 to 2022-08-13). The RMSE, MAE and Pearson correlation for each method across all states are reported in the violin plot. The methods (x-axis) are sorted based on their RMSE.

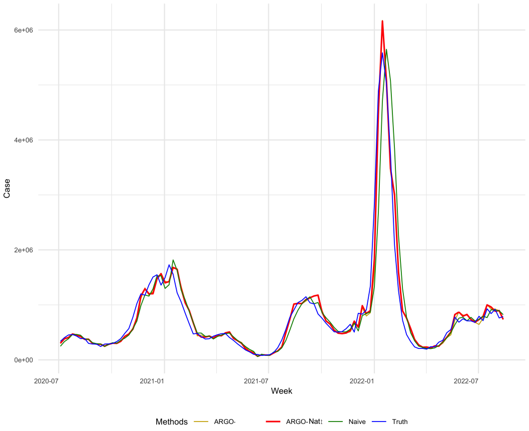

(a) 1 Week Ahead National Level Predictions

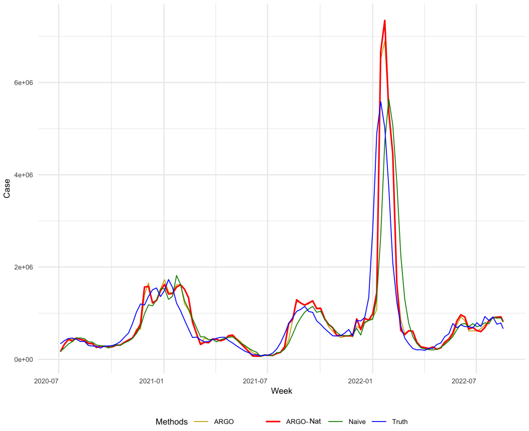

(b) 2 Weeks Ahead National Level Predictions

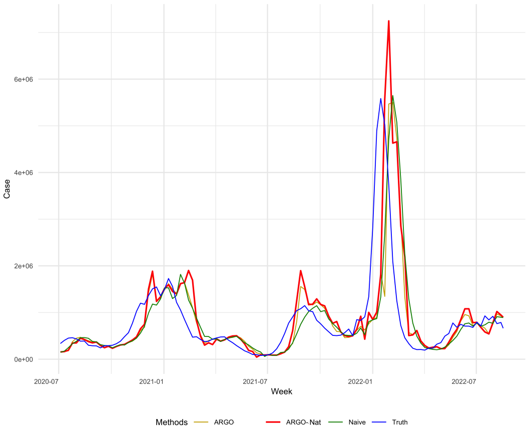

(c) 3 Weeks Ahead National Level Predictions

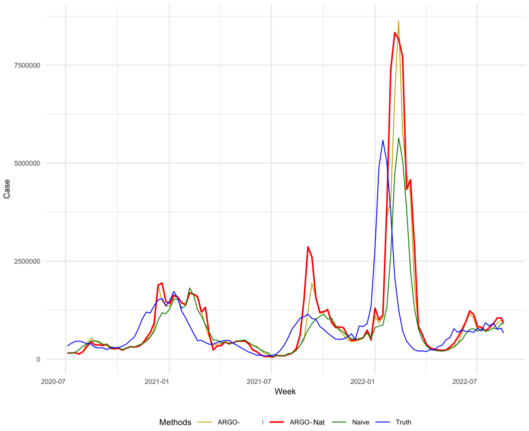

(d) 4 Weeks Ahead National Level Predictions

Figure S7: National level 1 to 4 weeks ahead COVID-19 weekly cases predictions from 2020-07-04 to 2022-08-13. The method included are single-disease ARGO (Ref [44]), ARGO-Nat, Naive (persistence), truth. Estimation results for 1 (top left), 2 (top right), 3 (bottom left), and 4 (bottom right) weeks ahead weekly cases. ARGO-Nat estimations (thick red), contrasting with the true COVID-19 cases from JHU dataset (blue) as well as the estimates from Ref [44] (gold), and Naive (green).

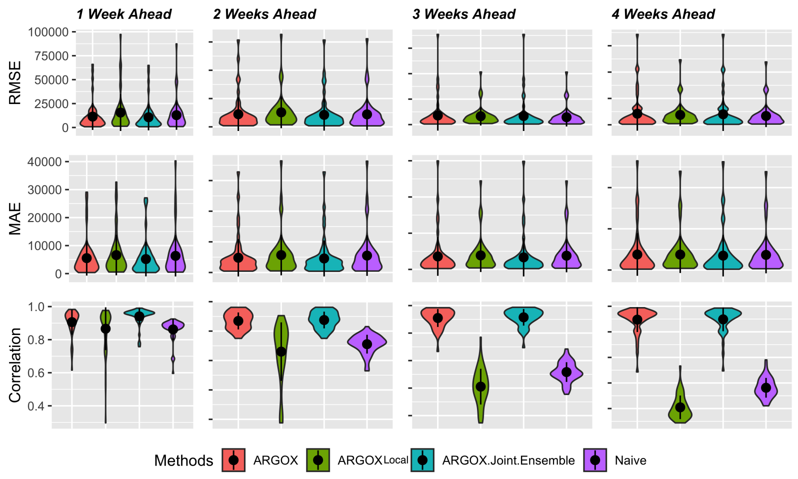

Figure S8: State Level Case Prediction Comparison in 3 Error Metrics. Comparison among different versions of our models' 1 to 4 weeks (from left to right) ahead U.S. states level weekly case predictions (from 2020-07-04 to 2022-08-13). The RMSE, MAE and Pearson correlation for each method across all states are reported in the violin plot. The methods (x-axis) are sorted based on their RMSE. Here, ARGOX denotes the previously proposed method [44].

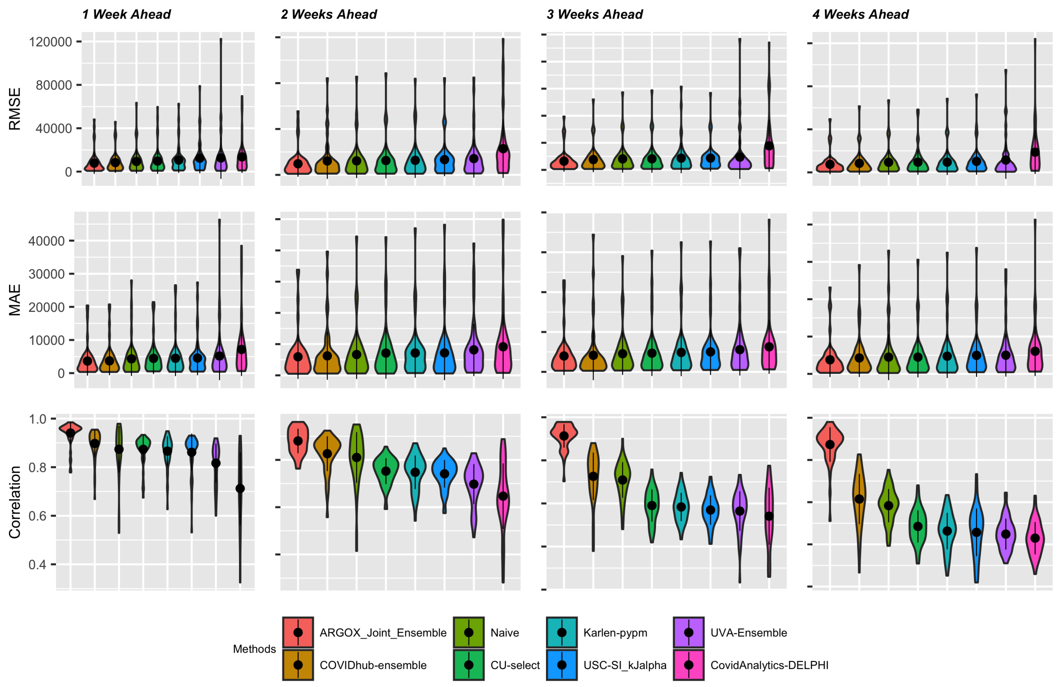

Figure S9: State Level Case Prediction Comparisons among different CDC published teams' 1 to 4 weeks (from left to right) ahead weekly deaths (from 2020-07-04 to 2022-08-13). The RMSE, MAE and Pearson correlation for each method across all states are reported in the violin plot. The methods (x-axis) are sorted based on their RMSE.

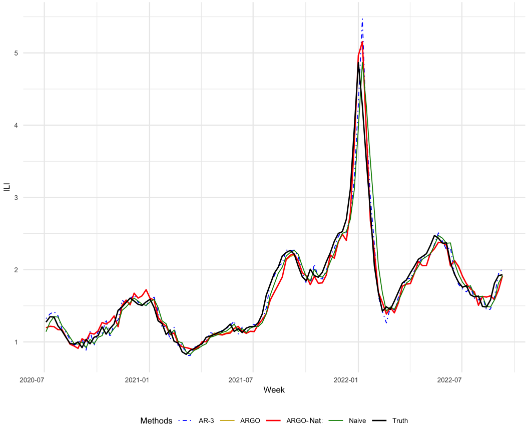

(a) 1 Week Ahead National Level Predictions

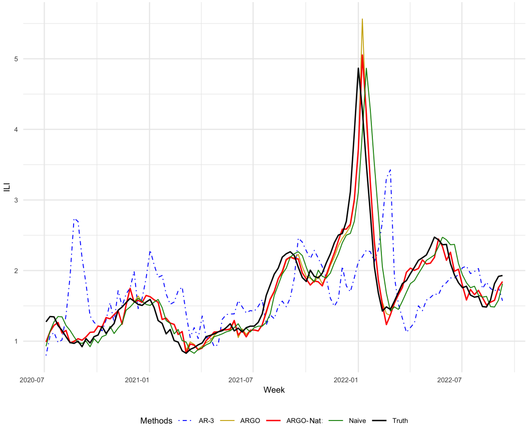

(b) 2 Weeks Ahead National Level Predictions

Figure S10: National level 1-2 weeks ahead %ILI predictions from 2020-07-04 to 2022-08-13. The method included are single-disease ARGO (Ref [22]), bi-disease ARGO-Nat, AR-3, Naive (persistence), truth. ARGO-Nat estimations (thick red), contrasting with the true %ILI (black) as well as the estimates from Ref [22] (gold), Naive (green), and AR-3 (blue).

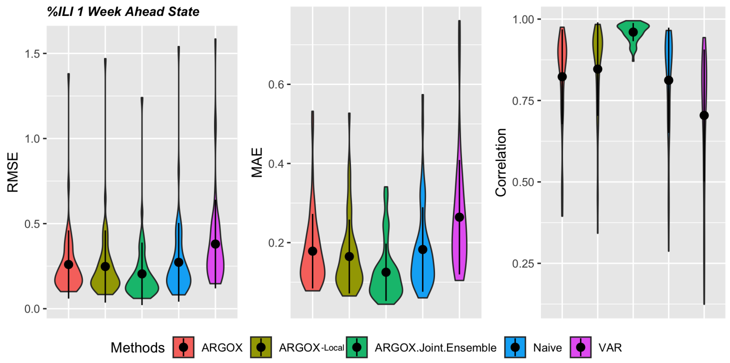

(a) 1 Week Ahead

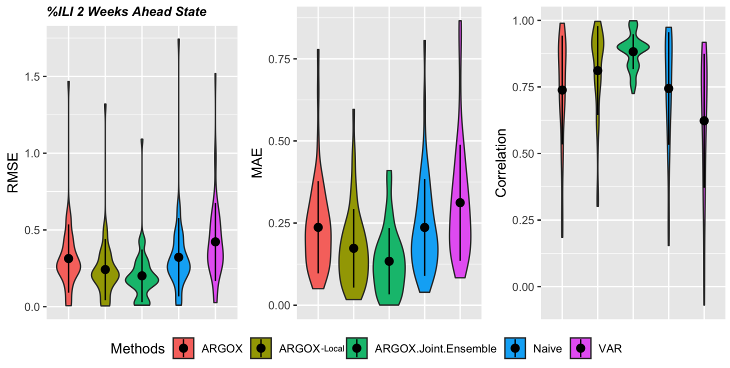

(b) 2 Weeks Ahead

Figure S11: State Level %ILI Prediction Comparison in 3 Error Metrics. Comparison among different versions of our models' and benchmark methods for 1 and 2 weeks ahead week ahead U.S. states level weekly %ILI predictions (from 2020-07-04 to 2022-08-13). The RMSE, MAE and Pearson correlation for each method across all states are reported in the violin plot. Here, ARGOX denotes Ref [24], ARGOX-Local and ARGOX-Joint-Ensemble are in the Methods section.

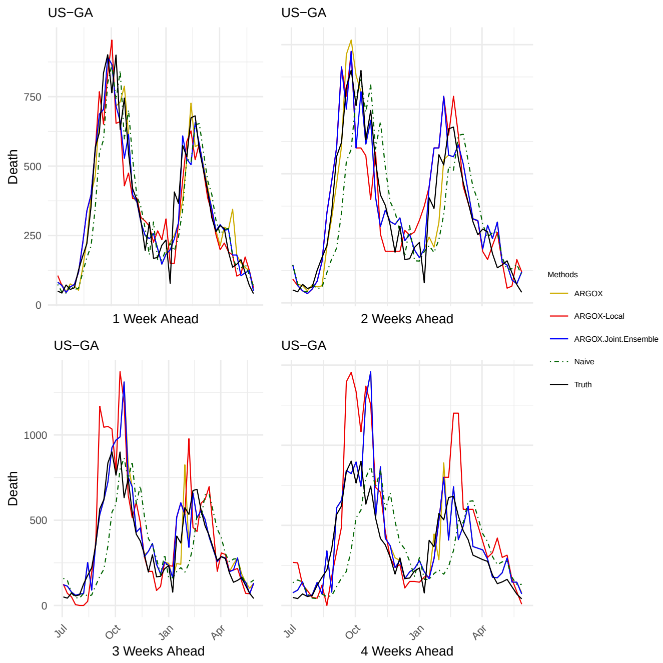

Figure S12: Plots of the COVID-19 1-4 weeks ahead estimates for Georgia (GA). ARGOX is Ref [44], and ARGOX-Local is proposed in this study (see Supplementary Materials). The plot shows the zoomed in period from July 2021 to August 2022.

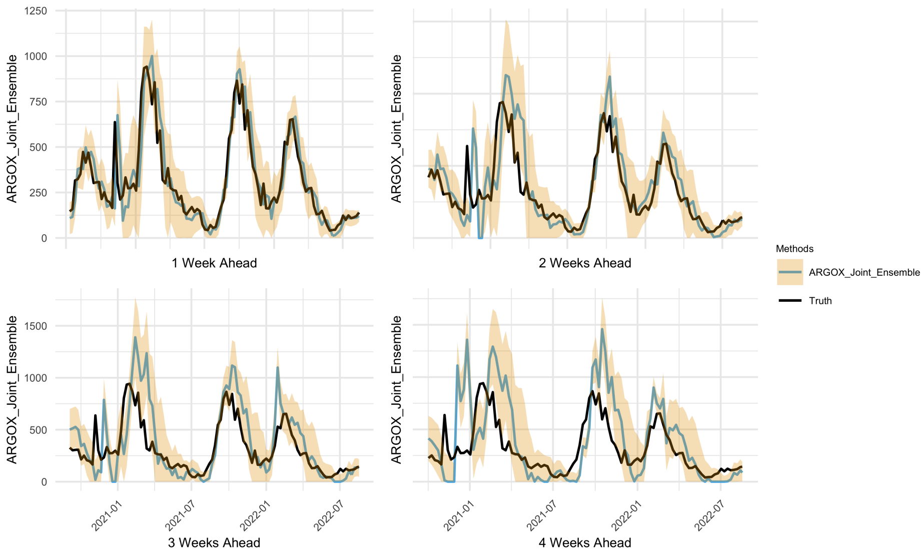

Figure S13: Plots of the COVID-19 1-4 weeks ahead point and probabilistic estimations for Georgia (GA). ARGOX-Joint-Ensemble's point estimation is shown in color "Blue" and prediction intervals is filled with color "Orange". Groundtruth (collected from JHU CSSE COVID-19 dataset [41]) is shown in color "Black". The plot shows the zoom in period from November 2020 to August 2022.

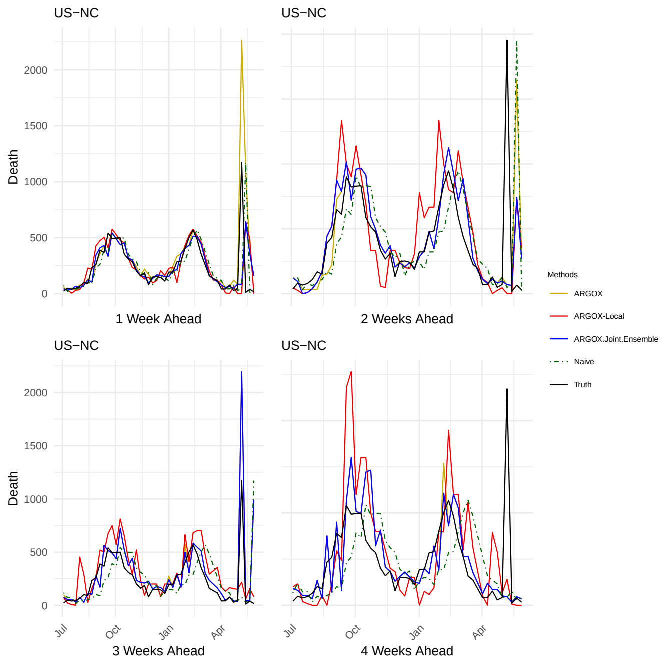

Figure S14: Plots of the COVID-19 1-4 weeks ahead estimates for North Carolina (NC). ARGOX is Ref [44], and ARGOX-Local is proposed in this study (see Supplementary Materials). The plot shows the zoomed in period from July 2021 to August 2022.

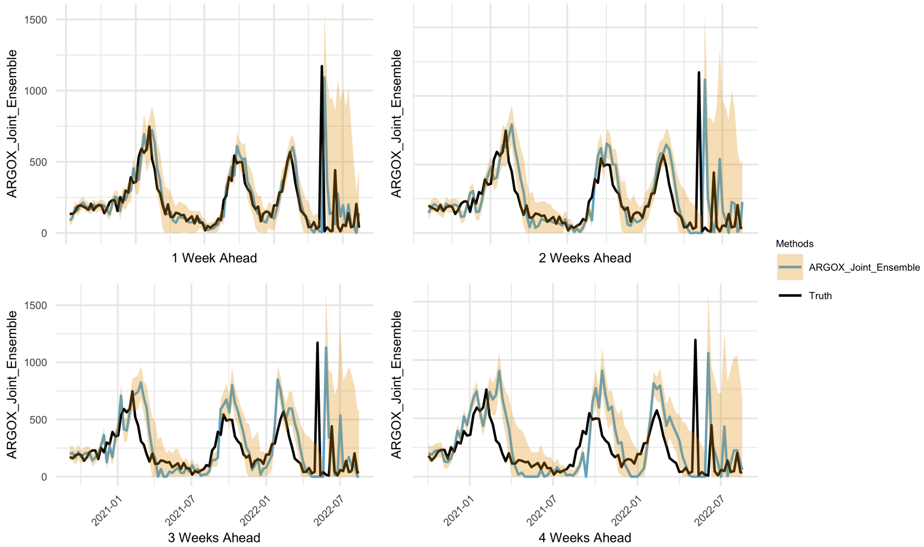

Figure S15: Plots of the COVID-19 1-4 weeks ahead point and probabilistic estimations for North Carolina (NC). ARGOX-Joint-Ensemble's point estimation is shown in color "Blue" and prediction intervals is filled with color "Orange". Groundtruth (collected from JHU CSSE COVID-19 dataset [41]) is shown in color "Black". The plot shows the zoom in period from November 2020 to August 2022.

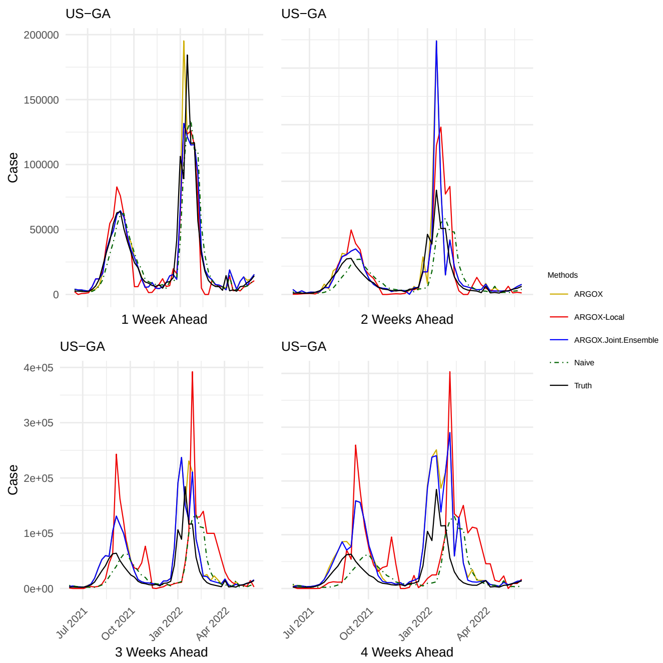

Figure S16: Plots of the COVID-19 1-4 weeks ahead estimates for Georgia (GA). ARGOX is Ref [44], and ARGOX-Local is proposed in this study (see Supplementary Materials). The plot shows the zoomed in period from July 2021 to August 2022.

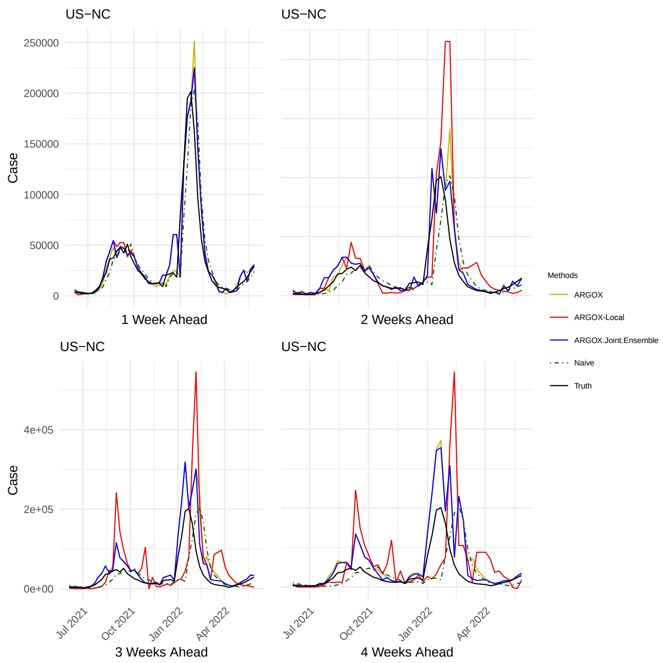

Figure S17: Plots of the COVID-19 1-4 weeks ahead estimates for North Carolina (NC). ARGOX is Ref [44], and ARGOX-Local is proposed in this study (see Supplementary Materials). The plot shows the zoomed in period from July 2021 to August 2022.

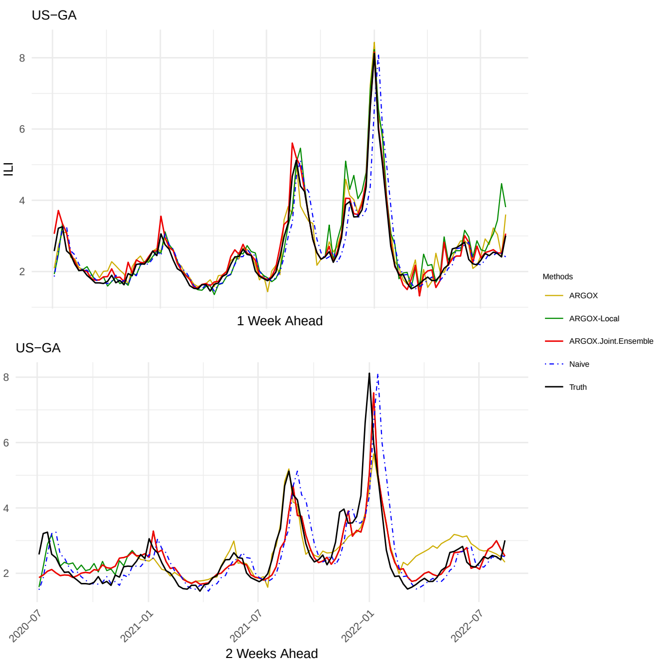

Figure S18: Plots of the %ILI 1-2 weeks ahead estimates for Georgia (GA). ARGOX is Ref [24] and ARGOX-Local is proposed in this study. The plot shows the entire evaluation period, from 2020-07-04 to 2022-08-13.

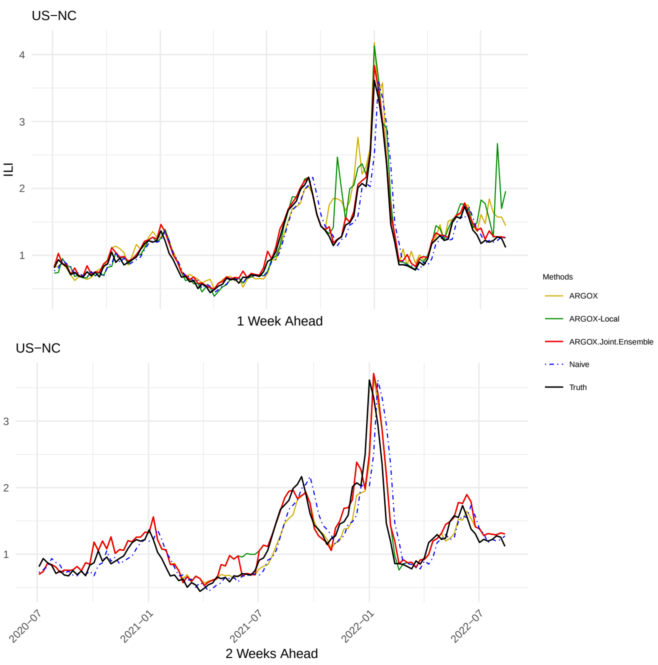

Figure S19: Plots of the %ILI 1-2 weeks estimates for North Carolina (NC). ARGOX is Ref [24] and ARGOX-Local is proposed in this study. The plot shows the entire evaluation period, from 2020-07-04 to 2022-08-13.

Supplementary Discussion

To further examine the ARGOX-Joint-Ensemble's performance gain from Ref [44] and ARGOX-Local in the state-level, we zoom in on two U.S. states (Georgia and North Carolina) and evaluate the forecasting performances of the three methods (see Table S16, S18, and Figure S12, S14). Since the %ILI trend is typically 2 to 3 weeks ahead of COVID-19 death trends (Figure 1) and each state's epidemic growth trend is more correlated with neighboring states than states further apart, ARGOX-Local, through exploiting the "correlation" between COVID-19 deaths and %ILI trends within neighboring states, is more robust during the increasing and peaking periods than single disease models (Ref [44]). In particular, ARGOX-Local is able to "foresee" the upcoming outbreaks sooner than ARGOX (Ref [44]) prior to the increasing periods from July 2021 to October 2021, from January 2022 to March 2022, as well as the peaking periods around early October 2021 and early February 2022, especially for 1 and 2 weeks ahead forecasts in GA (Figure S16) and NC (Figure S14). However, the additional ILI information could potentially inflate the ARGOX-Local estimations around the peaking periods, especially during early February 2022 (Omicron variant). Furthermore, as ILI signal gradually loses its predictive power when forecasting horizon extends to 3 and 4 weeks, ARGOX-Local could be misled by the deteriorated ILI signal, resulting in overshooting and delayed recovery during and after peaking periods (Figure S16, S14). Luckily, the ensemble framework is able to robustly select the best sub-model estimates among Ref [44] and ARGOX-Local for future 1-4 weeks predictions. Specifically, in Georgia, ARGOX-Joint-Ensemble selects ARGOX-Local during the increasing periods from July 2021 to October 2021 and from January 2022 to March 2022 almost all the time for 1 and 2 weeks ahead forecasts, while "fall back" to one of the other three single disease sub-models in Ref [44] during the decreasing periods from October 2021 to December 2021 and from March 2022 to May 2022 (Figure S16). In North Carolina, ARGOX-Joint-Ensemble's selection is similar, and it is also able to recognize the "best" sub-model. For example, during the increasing period from January 2022 to March 2022, for 2 weeks ahead forecasts, ARGOX-Local estimates the increasing trend too aggressively and therefore ARGOX-Joint-Ensemble selects the sub-models from Ref [44] instead. As ARGOX-Local's performances gradually deteriorates for 3 and 4 weeks ahead forecasts, ARGOX-Joint-Ensemble can quickly recover and select the alternative sub-models instead (Table S4 and Figure S12, S17). Other state's ARGOX-Local performances and ARGOX-Joint-Ensemble selection behaviors are similar to Georgia and North Carolina above. Therefore, ARGOX-Joint-Ensemble is able to uniformly outperform ARGOX-Local and Ref [44] on average across all the states (Table 1).

In additional to the point estimate's analysis and visualisations, we also illustrate the ARGOX-Joint-Ensemble's probabilistic predictions' performances in Georgia and North Carolina. Table S17 and S19 show the 1-4 weeks ahead Georgia and North Carolina's COVID-19 deaths prediction intervals' performance comparisons between ARGOX-Joint-Ensemble and COVIDhub-ensemble [34] (due to space limitations), through the two probabilistic error metrics: weighted interval score (WIS) and empirical coverage. Figure S13 and S15 visualize the ARGOX-Joint-Ensemble's point estimates (in color "Blue") against the groundtruth collected from JHU CSSE COVID-19 dataset [41] (in color "Black"), as well as ARGOX-Joint-Ensemble's prediction intervals (filled with color "Orange"), for 1-4 weeks ahead Georgia and North Carolina's COVID-19 death trends. COVIDhub-ensemble's [34] probabilistic predictions are omitted in the visualization for clarity. Through the robust selection of the best sub-model point estimates among Ref [44] and ARGOX-Local for 1-4 weeks ahead forecasts, ARGOX-Joint-Ensemble is able to produce reasonable probabilistic predictions for Georgia and North Carlina (Figure S13 and S15). Specifically, for 1-2 weeks head predictions, ARGOX-Joint-Ensemble's prediction intervals shows robust coverage in different rapidly changing dynamics, subject to the forecasts' and data uncertainty. For example, in Georgia during summer 2021 and winter 2021, the COVID-19 death trends exhibited rapid increases and decreases, and therefore ARGOX-Joint-Ensemble produced wider prediction intervals as the uncertainties in the predictions increased. On the other hand, ARGOX-Joint-Ensemble produced narrower prediction intervals from July 2021 to November 2021, and from April 2022 to July 2022, as the COVID-19 death trends was strictly increasing or decreasing, and the ensemble framework is more certain towards the predictions. Other states' prediction intervals' performances exhibit similar patterns as Georgia and North Carolina.
