# Supplementary material for: Joint COVID-19 and influenza-like illness forecasts in the United States using internet search information
Source: Commun Med (Lond). 2023 Mar 24;3:39. doi: 10.1038/s43856-023-00272-2 (PMC10038385; doi:10.1038/s43856-023-00272-2)
Supplement: Supplementary file 5 — Reporting Summary [file 43856_2023_272_MOESM5_ESM.pdf]

nature portfolio

Corresponding author(s): Shihao Yang

Last updated by author(s): Feb 10, 2023

Reporting Summary

Nature Portfolio wishes to improve the reproducibility of the work that we publish. This form provides structure for consistency and transparency in reporting. For further information on Nature Portfolio policies, see our Editorial Policies and the Editorial Policy Checklist.

Statistics

For all statistical analyses, confirm that the following items are present in the figure legend, table legend, main text, or Methods section.

n/a	Confirmed	
☒	☐	The exact sample size (*n*) for each experimental group/condition, given as a discrete number and unit of measurement
☐	☒	A statement on whether measurements were taken from distinct samples or whether the same sample was measured repeatedly
☐	☒	The statistical test(s) used AND whether they are one- or two-sided<br>*Only common tests should be described solely by name; describe more complex techniques in the Methods section.*
☐	☒	A description of all covariates tested
☐	☒	A description of any assumptions or corrections, such as tests of normality and adjustment for multiple comparisons
☐	☒	A full description of the statistical parameters including central tendency (e.g. means) or other basic estimates (e.g. regression coefficient) AND variation (e.g. standard deviation) or associated estimates of uncertainty (e.g. confidence intervals)
☐	☒	For null hypothesis testing, the test statistic (e.g. *F*, *t*, *r*) with confidence intervals, effect sizes, degrees of freedom and *P* value noted<br>*Give P values as exact values whenever suitable.*
☒	☐	For Bayesian analysis, information on the choice of priors and Markov chain Monte Carlo settings
☒	☐	For hierarchical and complex designs, identification of the appropriate level for tests and full reporting of outcomes
☒	☐	Estimates of effect sizes (e.g. Cohen's *d*, Pearson's *r*), indicating how they were calculated

Our web collection on statistics for biologists contains articles on many of the points above.

Software and code

Policy information about availability of computer code

Data collection	The online search data sets that support the findings of this study are publicly available from Google Trends, and also deposited to Harvard Dataverse (DOI: 10.7910/DVN/PGNBAX), that is publicly available. We use standard R software environment (version 3.6.1) to extract and preprocess the above data. All detailed pre-processing and collection code can be found in the publicly available Github repo: https://github.com/stevenmsm/Joint-COVID-19-and-Influenza-Forecasts-in-the-United-States
Data analysis	We use standard R software environment (version 3.6.1) to conduct all data analysis. Detailed R code are all provided in the publicly available Github repo: https://github.com/stevenmsm/Joint-COVID-19-and-Influenza-Forecasts-in-the-United-States

For manuscripts utilizing custom algorithms or software that are central to the research but not yet described in published literature, software must be made available to editors and reviewers. We strongly encourage code deposition in a community repository (e.g. GitHub). See the Nature Portfolio guidelines for submitting code & software for further information.

Data

Policy information about availability of data

All manuscripts must include a data availability statement. This statement should provide the following information, where applicable:

- Accession codes, unique identifiers, or web links for publicly available datasets
- A description of any restrictions on data availability
- For clinical datasets or third party data, please ensure that the statement adheres to our policy

The online search data sets that support the findings of this study are publicly available from Google Trends , and also deposited to Harvard Dataverse (DOI: 10.7910/DVN/PGNBAX). The downloading date of COVID-19 related Google search data is 2022-08-14 and the downloading date of %ILI related Google search data is 2022-09-11. The COVID-19 confirmed cases and deaths data are publicly available from JHU CSSE COVID-19 dataset, while hospitalization data are publicly available from Health and Human Services and the official CDC Forecast Hub. %ILI data are publicly available from CDC.

Human research participants

Policy information about studies involving human research participants and Sex and Gender in Research.

Reporting on sex and gender	Not applicable
Population characteristics	Not applicable
Recruitment	Not applicable
Ethics oversight	Not applicable

Note that full information on the approval of the study protocol must also be provided in the manuscript.

Field-specific reporting

Please select the one below that is the best fit for your research. If you are not sure, read the appropriate sections before making your selection.

☒ Life sciences ☐ Behavioural & social sciences ☐ Ecological, evolutionary & environmental sciences

For a reference copy of the document with all sections, see nature.com/documents/nr-reporting-summary-flat.pdf

Life sciences study design

All studies must disclose on these points even when the disclosure is negative.

Sample size	No sample-size calculation is performed. The study focus on infectious disease forecasting task and the population of interest is U.S. national-level and state-level population.
Data exclusions	No data were excluded from the analysis.
Replication	All attempts are replication were successful.
Randomization	Not relevant. All data were publicly available provided by different health-care organization. No randomization is done in this study.
Blinding	Not relevant, as there are no treatment or control assignments in this study.

Reporting for specific materials, systems and methods

We require information from authors about some types of materials, experimental systems and methods used in many studies. Here, indicate whether each material, system or method listed is relevant to your study. If you are not sure if a list item applies to your research, read the appropriate section before selecting a response.

Materials & experimental systems

n/a	Involved in the study
☒	☐ Antibodies
☒	☐ Eukaryotic cell lines
☒	☐ Palaeontology and archaeology
☒	☐ Animals and other organisms
☒	☐ Clinical data
☒	☐ Dual use research of concern

Methods

n/a	Involved in the study
☒	☐ ChIP-seq
☒	☐ Flow cytometry
☒	☐ MRI-based neuroimaging
